# Quantitative Proteomic Analysis Reveals Molecular Adaptations in the Hippocampal Synaptic Active Zone of Chronic Mild Stress-Unsusceptible Rats

**DOI:** 10.1093/ijnp/pyv100

**Published:** 2015-09-12

**Authors:** Jian Zhou, Zhao Liu, Jia Yu, Xin Han, Songhua Fan, Weihua Shao, Jianjun Chen, Rui Qiao, Peng Xie

**Affiliations:** Institute of Neuroscience and the Collaborative Innovation Center for Brain Science, Chongqing Medical University, Chongqing, China (Drs Zhou, Liu, Yu, Han, Fan, Shao, Chen, Qiao, and Xie); Chongqing Key Laboratory of Neurobiology, Chongqing, China (Drs Zhou, Liu, Yu, Han, Fan, Shao, Chen, Qiao, and Xie); Department of Neurology, the First Affiliated Hospital of Chongqing Medical University, Chongqing, China (Drs Liu, Han, Fan, Shao, and Xie).

**Keywords:** major depressive disorder, chronic mild stress, hippocampus, synapse, proteomics

## Abstract

**Background:**

While stressful events are recognized as an important cause of major depressive disorder, some individuals exposed to life stressors maintain normal psychological functioning. The molecular mechanism(s) underlying this phenomenon remain unclear. Abnormal transmission and plasticity of hippocampal synapses have been implied to play a key role in the pathoetiology of major depressive disorder.

**Methods:**

A chronic mild stress protocol was applied to separate susceptible and unsusceptible rat subpopulations. Proteomic analysis using an isobaric tag for relative and absolute quantitation coupled with tandem mass spectrometry was performed to identify differential proteins in enriched hippocampal synaptic junction preparations.

**Results:**

A total of 4318 proteins were quantified, and 89 membrane proteins were present in differential amounts. Of these, SynaptomeDB identified 81 (91%) having a synapse-specific localization. The unbiased profiles identified several candidate proteins within the synaptic junction that may be associated with stress vulnerability or insusceptibility. Subsequent functional categorization revealed that protein systems particularly involved in membrane trafficking at the synaptic active zone exhibited a positive strain as potential molecular adaptations in the unsusceptible rats. Moreover, through STRING and immunoblotting analysis, membrane-associated GTP-bound Rab3a and Munc18-1 appear to coregulate syntaxin-1/SNAP25/VAMP2 assembly at the hippocampal presynaptic active zone of unsusceptible rats, facilitating SNARE-mediated membrane fusion and neurotransmitter release, and may be part of a stress-protection mechanism in actively maintaining an emotional homeostasis.

**Conclusions:**

The present results support the concept that there is a range of potential protein adaptations in the hippocampal synaptic active zone of unsusceptible rats, revealing new investigative targets that may contribute to a better understanding of stress insusceptibility.

## Introduction

Major depressive disorder (MDD) is a debilitating psychiatric mood disorder with a lifetime prevalence of 16% that contributes to increased rates of disability and suicide (Kessler et al., 2003; Xu et al., 2012). Stressful life events are the most significant priming factor in the etiology of MDD (Kendler et al., 1999). However, some individuals are able to successfully cope with acute stress or more prolonged chronic forms of adversity (Franklin et al., 2012). These ‘‘resilient’’ individuals display traits such as cognitive flexibility and optimism (Fleshner et al., 2011). Resiliency is not merely a lack of stress susceptibility; it is an active and adaptive psychological and physiological stress response or “psychobiological allostasis” (Feder et al., 2009; Franklin et al., 2012; Russo et al., 2012). This resilient phenotype is important in understanding the underlying biological processes associated with stress susceptibility and resiliency (Charney, 2004; Taliaz et al., 2011). However, the neural substrates and molecular mechanisms that mediate resistance to the deleterious effects of stress remain unclear (Krishnan et al., 2007; Friedman et al., 2014).

As one of most malleable brain regions targeted by stress stimulation (McEwen et al., 2012), the hippocampus shoulders the responsibility of balancing function and vulnerability to stress damage by adaptive neuron dendritic remodeling, such as growth and shrinkage of dendritic trees and spines (Sousa et al., 2000; McEwen, 2010). This hippocampal dendritic or spine remodeling is dysregulated in stress-induced depression and stress resilience (Duman and Aghajanian, 2012; Gourley et al., 2013). In animal models, chronic stress reduces hippocampal neurogenesis (Kempermann and Kronenberg, 2003; Paizanis et al., 2007; Kim et al., 2013) and the complexity of dendritic arbors while impairing function in assays of hippocampal dependent spatial memory (Nestler et al., 2002; Bannerman et al., 2014) and long-term potentiation (Bannerman et al., 2012). Using noninvasive magnetic resonance methods, hippocampal microstructural variations have been found in both chronic mild stress (CMS)-exposed anhedonic and resilient rats. In contrast, morphological and metabolic changes of the hippocampus allows discrimination of these 2 subtypes, suggesting that stress induces hippocampal reorganization through different pathways (Delgado y Palacios et al., 2011).

Generally, the signs of hippocampal reorganization were sought by examining the plasticity of dendrites and synapses (Leuner and Gould, 2010). Since memories are believed to be stored and maintained in hippocampal synapses (Bliss and Collingridge, 1993), synaptic plasticity is thought to be the cellular mechanism for learning and memory that guides the behavior of an organism (Bannerman et al., 2014). long-term potentiation of synaptic transmission remains the most widely studied example of synaptic plasticity, especially in the hippocampus (Holderbach et al., 2007; Maggio and Segal, 2011; Kamal et al., 2014). Moreover, dysregulation of hippocampal synaptic plasticity has also been implicated in a variety of psychiatric disorders (Bannerman et al., 2014). In animals, chronic stress severely disturbs synaptic plasticity. For example, changes in the strength or efficacy of synaptic transmission result in hippocampal structural and functional incoordination (Sousa et al., 2000; Kim et al., 2006; Holderbach et al., 2007; Pittenger and Duman, 2008). In humans, the sustained increase of hippocampal excitatory synaptic transmission following stress may underlie the dendritic remodeling and volumetric shrinkage associated with stress-related pathologies (Maras and Baram, 2012; Sanacora et al., 2012). Alternatively, synaptic plasticity allows the organism to adapt to a constant stress environment even under high-stress conditions, implying that a strong adaptive regulation program over stress pathways may be exerted (Christoffel et al., 2011; Delgado y Palacios et al., 2011). However, the specific proteins and genes that are required for stress-related synaptic remodeling and adaptation remain unclear (Christoffel et al., 2011). Uncovering these numerous molecular events is critical to both the understanding of the etiology of MDD as well as the anhedonic or resilient nature.

To study the stress-related molecular events intrinsic to the hippocampal synapse, direct approaches to quantitatively address the subtype-specific synaptic proteome are necessary. This can be achieved by biochemically enriching synaptic junctions that are taken from active zone-associated electron-dense structures (Phillips et al., 2001). Such an approach has the important advantage of revealing localized events that are otherwise hidden in the complexity of molecular changes that occur in other subcellular compartments (Phillips et al., 2001; Abul-Husn et al., 2009; Dahlhaus et al., 2011). Although some synaptic molecules have been intermittently studied by molecular detection techniques at the tissue level from various stressed systems (Muller et al., 2011; Henningsen et al., 2012; Marrocco et al., 2012; Duric et al., 2013; Wang et al., 2014), the distinct picture of these components, particularly at the synaptic active zone, remains elusive.

Here, we carried out nonhypothesis-driven, large-scale proteomic analyses using isobaric tag for relative and absolute quantitation (iTRAQ) coupled with tandem mass spectrometry. We utilized this approach to investigate quantitative changes of proteins from the enriched synaptic junction preparations in a CMS model of depression. This depressive model generates both susceptible and unsusceptible subpopulations, reflecting the 2 hedonic responses to CMS. Our synaptic proteome profiles identified several potential molecular adaptations within the synaptic active zone that may be related to stress vulnerability or insusceptibility and represent differences in important active biological processes specific to stress vulnerability and stress coping strategies.

## Materials and Methods

A detailed description of the materials and methods used in this study is provided in the supplementary Methods.

### Animal Subjects and CMS Protocol

**Figure 1.**
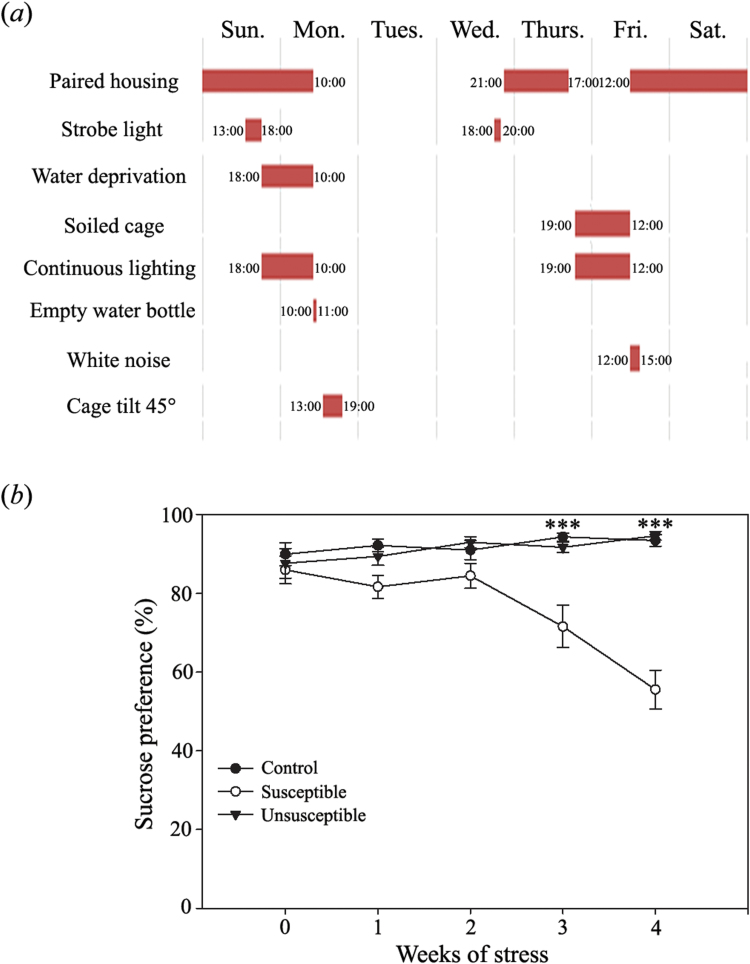
Chronic mild stress (CMS) schedule and sucrose preference test. (*a*) Time and length of stressors used in the CMS procedure. (*b*) Sucrose preference during the CMS protocol. By the third week of CMS procedure, sucrose preference was significantly decreased in the susceptible rats compared with the control and unsusceptible rats (n=19). This relationship continued into the fourth week of CMS as well. ***P<.001.

Male Sprague-Dawley rats from the Animal Facility of Chongqing Medical University were used. The CMS protocol was then performed according to previously described methods (Willner et al., 1987; Grippo et al., 2006; Hu et al., 2013; Yang et al., 2013). Details of the procedure including time and length of stressors are presented in Figure 1a.

### Behavioral Experiments

The sucrose preference test (Hu et al., 2013), open-field test (OFT) (Yang et al., 2013), and forced swimming test (FST) (Porsolt et al., 1977) were conducted as previously described.

### Sample Preparation, iTRAQ Labeling, and SCX Fractionation

Hippocampal synaptic junction-enriched fractions were obtained as previously reported (Phillips et al., 2001; Abul-Husn et al., 2009; Hu et al., 2013). Then, the synaptic junctional proteins were extracted, digested (Wisniewski et al., 2009), and labeled by iTRAQ-4plex reagents (Applied Biosystems) according to the manufacturer’s instruction. Labeled peptides were combined and fractionated by strong cation exchange (SCX) chromatography.

### Liquid Chromatography-Tandem Mass Spectrometry (LC-MS/MS) and Data Analysis

As described in our previous study (Zhan et al., 2014), the SCX fractions were analyzed using a TripleTOF 5600 mass spectrometer (AB SCIEX) equipped with a splitless nanoLC-Ultra 2D plus system and a cHiPLC Nanoflex microchip system. Subsequent protein identification and iTRAQ quantitation were performed with ProteinPilot 4.5 software (AB SCIEX) using the Paragon algorithm (4.5.0.0.1654) as the search engine. Identified proteins were grouped by the ProGroup algorithm to minimize redundancy. For quantitative analysis, observed proteins with iTRAQ ratios of >1.2 and <0.83 were considered to be differentially expressed, as used in previous studies (Lin et al., 2013; Zhang et al., 2014; Wang et al., 2015). All raw and metadata of the proteome have been deposited to the ProteomeXchange with identifier PXD002540.

### Bioinformatics Analysis

The gene ontology (GO) annotations (including subcellular location and function) of the identified differential proteins were obtained from the UniProt Knowledgebase and the DAVID Database (Huang da et al., 2009). More detailed descriptions of these differential membrane proteins were derived from SynaptomeDB (Pirooznia et al., 2012). Furthermore, the identified proteins involved in membrane trafficking were mapped to the protein interaction network, and STRING was used to qualify the physical and functional interactions of these proteins (Zhou et al., 2012a).

### Antibodies and Western-Blot Analyses

For Western blot, the procedures of electrophoresis, transfer, and immunodetection were performed according to our previous study (Xu et al., 2012; Hu et al., 2013). The primary antibodies used were as follows: antibody for the Ras-related protein Rab-3A (Rab3a, ab3335, 1:2000); syntaxin-binding protein 1 (Stxbp1, Munc18-1, ab124920, 1:4000); synapsin-1 (Syn1, ab18814, 1:1000); syntaxin-1A (Stx1a, ab41453, 1:3000); synaptosomal-associated protein 25 (SNAP25, ab5666, 1:4000); vesicle-associated membrane protein 2 (VAMP2, ab3347, 1:2000); synaptophysin (ab52636, 1:1000) (all purchased from Abcam); synaptotagmin 1 (Syt1, Millipore MAB5200, 1:1000); syntaxin-1B (Stx1b, Synaptic Systems 110402, 1:1000); and PSD95 (CST #3450, 1:1000). Horseradish peroxidase-conjugated anti-mouse and anti-rabbit IgG (purchased from Bio-Rad, dilution 1:15000) were used as secondary antibodies. After immunodetection, the intensity of the immunostained bands were normalized for the total protein intensities measured by Coomassie blue from the same blot (Van den Oever et al., 2008; Counotte et al., 2010). The images were subjected to densitometric analysis performed using Quantity One software (Bio-Rad).

### Statistical Analysis

The sucrose preference test, OFT, and FST data were analyzed using SPSS 16.0, as described in our previous study (Hu et al., 2013; Yang et al., 2013). Meanwhile, the data from Western blots of protein expression were compared using Student’s *t* tests. The level of statistical significance for all analysis was set at *P*<.05. Statistics were presented as means ±SE.

## RESULTS

### CMS-Induced Behavioral Assessment

Here, sucrose preference was applied to assess the stress-induced anhedonic-like (susceptible) and stress-resilient (unsusceptible) behavior of rats. A subset of the controls, susceptible, and unsusceptible rats (n=19 in each group), were used in the following analysis. Repeated measurement ANOVA showed that, for sucrose preference, the impact of the treatment factor (F(2, 54)=6.745, *P*<.001) and interaction between time and treatment (F(8, 216)=8.176, *P<.*001) were significant. MANOVA indicated that 3-week exposure to CMS resulted in significantly decreased sucrose preference of the susceptible group when compared with the control and unsusceptible groups (F(2, 54)=14.725, *P<.*001). This difference remained significant following 4 weeks of CMS (F(2, 54)=53.253, *P<.*001) as shown in Figure 1b.

**Figure 2.**
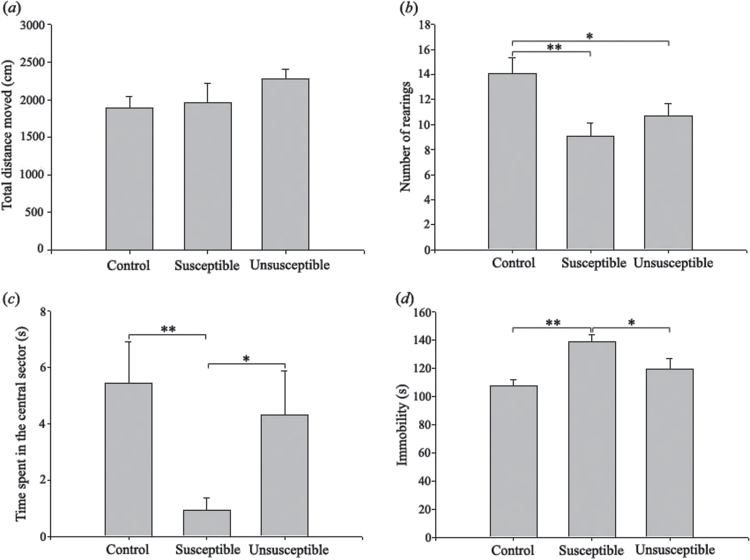
Results of behavioral testing. Comparisons of (*a*) total distance moved, (*b*) number of rearings, (*c*) time spent in the central sector of the open-field test (OFT), and (*d*) immobility times in the forced swimming test (FST) between the control, susceptible, and unsusceptible rats are shown. *P<.05, **P<.01.

In OFT, the CMS protocol did not alter locomotor activity (Figure 2a). The rearing number was significantly decreased in susceptible and unsusceptible rats relative to controls (*P<.*01 and *P<.*05, respectively), indicating decreased exploratory behavior in these groups (Figure 2b). Time spent in the central sector was used to reflect the degree of anxiety (ie, animals displaying higher activity levels in the center of the arena were defined as less anxious). Compared with the control and unsusceptible animals, the susceptible animals spent a shorter amount of time in the center square (*P<.*01 and *P<.*05, respectively), indicating higher anxiety in this population (Figure 2c). In FST, immobility time was significantly elevated in the susceptible rats as compared with the control and the unsusceptible groups (*P<.*01 and *P<.*05, respectively). There was no significant difference between the control and unsusceptible groups, indicating that the increase in immobility time in chronically stressed rats is related to anhedonic status (Figure 2d).

### Quantitative Proteomic Analysis of Hippocampal Synaptic Junctions

**Figure 3.**
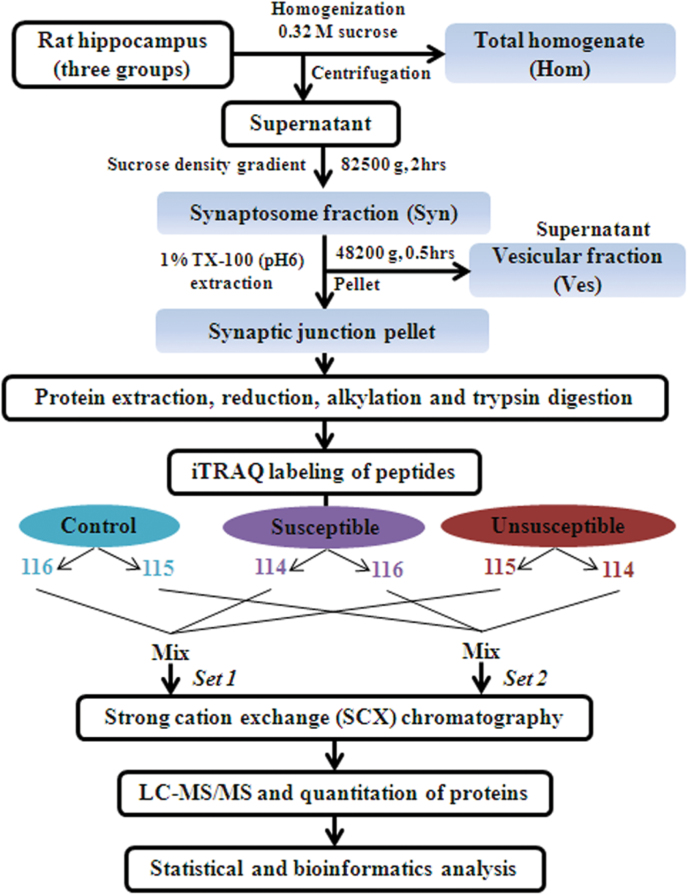
Outline of the sample preparation and isobaric tag for relative and absolute quantitation (iTRAQ) labeling procedures. Rat hippocampi were dissected from the 3 groups (control, susceptible, and unsusceptible). The tissue was subject to biochemical fractionation to obtain total homogenate (Hom), synaptosome (Syn), and synaptic vesicular (Ves) fractions. iTRAQ labeling was performed on the synaptic junction preparation. Two sets of biological replicate samples were analyzed using 4-plex iTRAQ reagents. Peptides from controls were labeled with iTRAQ reagent having 115 and 116 reporters, peptides from the susceptible group were labeled with iTRAQ reagent having 116 and 114 reporters, and peptides from the unsusceptible group were labeled with iTRAQ reagent having 114 and 115 reporters. After labeling, peptides from all 6 samples were separately combined and fractionated by strong cation exchange (SCX) chromatography. Each fraction was then analyzed by LC-MS/MS on a Triple TOF 5600 mass spectrometer.

**Figure 4.**
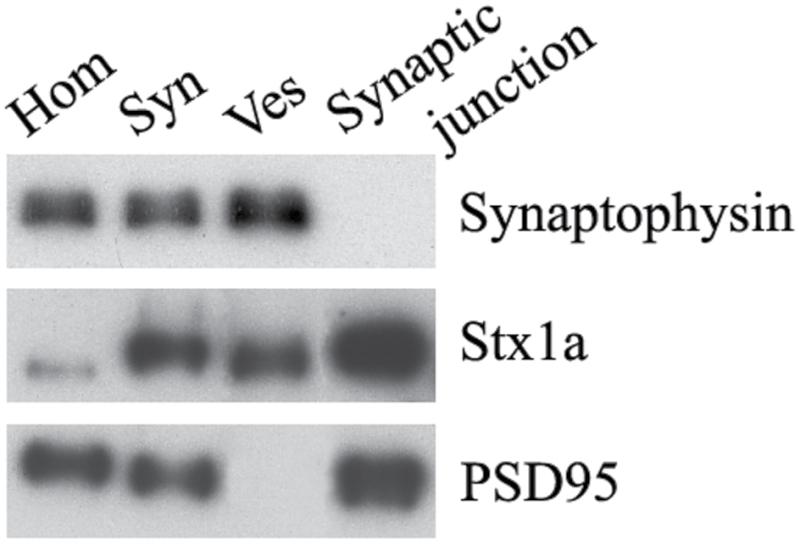
Western-blot analysis of the 4 fractions (Hom, Syn, Ves, and synaptic junction) obtained by biochemical fractionation. The fractions were analyzed using antibodies against synaptophysin (synaptic vesicle marker), Stx1a (synaptic active zone marker), and PSD95 (postsynaptic density marker).

**Figure 5.**
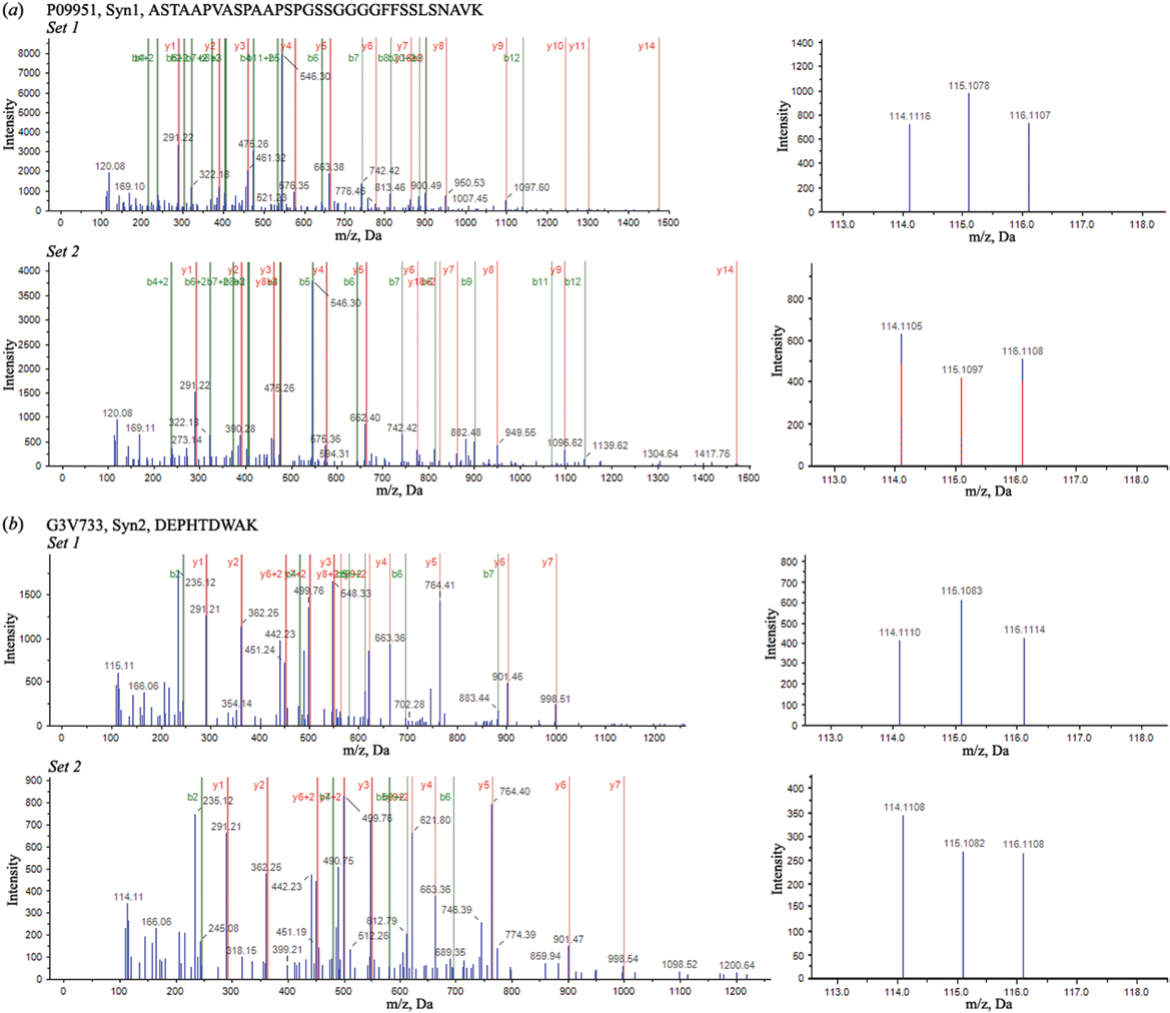
MS/MS spectra of peptides with their reporter ions for representative differentially expressed proteins in datasets 1 and 2. (*a*) MS/MS spectrum of a representative peptide (ASTAAPVASPAAPSPGSSGGGGFFSSLSNAVK) from Syn1 and corresponding spectrum showing relative intensity of reporter ions. (*b*) MS/MS spectrum of a representative peptide (DEPHTDWAK) from Syn2 and corresponding spectrum of reporter ions.

To identify differentially expressed proteins involved in mediating synaptic plasticity and neurotransmission in the hippocampus, we performed quantitative proteomics using an iTRAQ-based shotgun quantitation approach on fractions containing synaptic junctions derived from the hippocampi of groups of rats subjected to CMS conditions (Zhan et al., 2014). An overview of the sample preparation and iTRAQ workflow conducted is shown in Figure 3. Rat hippocampi tissue was subject to biochemical fractionation to obtain total homogenate (Hom), synaptosome (Syn), synaptic vesicular (Ves), and synaptic junction fractions. The resulting 4 fractions were analyzed to confirm expected enrichment by Western blot for appropriate marker proteins. As expected, the Ves fraction was enriched with the synaptic vesicle protein synaptophysin and free of PSD95, while the synaptic active zone protein Stx1a was mainly located in the synaptic junction fraction, demonstrating that our fractionation was effective (Figure 4). For quantitative proteomic analysis, iTRAQ labeling was performed on the synaptic junction preparation. After SCX fractionation and subsequent LC-MS/MS analysis, all protein and peptide identifications were obtained using the ProteinPilot search engine. The detailed protein identification and quantitation from 2 independent biological replicates are listed in supplementary Table 1. For all peptide matches with 95% confidence, the number of iTRAQ-labeled N-termini and lysines was compared with the total number of peptide N-termini and lysines (Pierce et al., 2008; Liu et al., 2009). The iTRAQ labeling efficiency was estimated to be 99.1% and 98.7% in datasets 1 and 2, respectively. A summary of the protein identification results is presented in Table 1. A total of 4562 distinct proteins was identified with at least 1 unique peptide and an estimated false discovery rate of 1% (2931 with more than 2 unique peptides); of these, 2620 (57%) were identified in both sets and 4318 proteins were quantified with 2344 (54%) overlapping in the replicate data sets. For further analysis, the proteins with <3 unique peptides (95% confidence) or error factor >2.0 were eliminated in the overlapping quantified proteins (Vegh et al., 2012). The exclusion criteria resulted in 1671 quantified proteins (see supplementary Table 2). The change in relative concentration of any given protein for the susceptible and unsusceptible groups relative to controls was obtained from the iTRAQ 4-plex reporter ion ratios by calculating a weighted average of all the confidently identified peptides assigned to any given protein. iTRAQ reporter ratios of 1.2 and 0.83 were set as the cut-off of protein changes (Lin et al., 2013; Zhang et al., 2014; Wang et al., 2015). Together with the 2 datasets, a total of 121 significantly altered proteins hits (either down- or upregulated) were statistically screened out for the respective sample comparisons. MS/MS spectra along with reporter ions of peptides belonging to representative proteins were shown in Figure 5.

**Table 1.** Comparison of the Two Sets of Biological Replicate Samples

Summary Data	Set 1	Set 2
Total number of protein IDs^*a*^	3691	3491
Number of unique proteins from each set^*b*^	1071	871
Combined distinct protein IDs (total/overlap) from the 2 sets	4562/2620	
Reproducibility of protein IDs in the 2 sets	71.0%	75.1%
Total proteins with iTRAQ ratio	3625 (98.2%)	3037 (87.0%)
Unique proteins with iTRAQ ratio from each set	1281	693
Combined distinct protein IDs with iTRAQ ratio (total/overlap) from the 2 sets	4318/2344	
Reproducibility of protein IDs with iTRAQ ratio in 2 sets	64.7%	77.2%
Total peptides/unique peptides	125708/26528 (21.1%)	106165/24160 (22.8%)
Unique peptides identified in only 1 set	10519	8151
Combined unique peptides (total/overlap) from the 2 sets	34679/16009	

Abbreviation: iTRAQ, isobaric tag for relative and absolute quantitation.

^*a*^The total number of protein IDs indicates the total protein IDs identified based on at least one unique peptide in the 2 sets of biological replicate samples.

^*b*^The number of unique proteins from each set denotes the number of protein IDs exclusively identified from each of the 2 sets.

### Identity Categorization of Differentially Expressed Proteins

**Figure 6.**
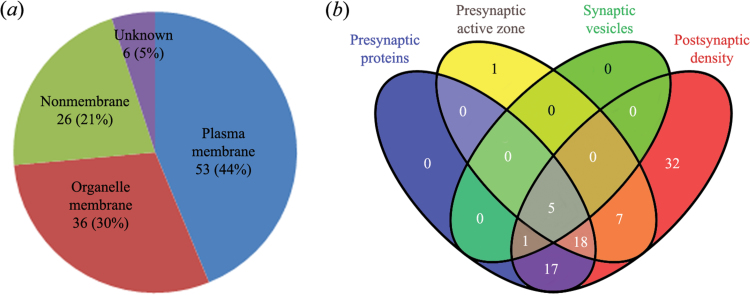
Localization analysis of differentially expressed proteins. (*a*) Pie chart showing the distribution of the differentially expressed proteins across the cellular compartments based on gene ontology (GO) annotations from the UniProt/DAVID database. (*b*) Venn diagram showing the detailed synaptic localization of the differentially expressed membrane-associated proteins based on the SynaptomeDB search.

The accession numbers of the 121 differential proteins identified from the susceptible and unsusceptible groups were uploaded into the UniProt and DAVID database for categorization of subcellular localization based on their GO annotations. In terms of cellular compartment, the majority of the differential proteins were membrane-associated components, of which 53 (44%) were localized to the plasma membrane and 36 (30%) were classified as organelle membrane proteins (Figure 6a).

To help focus on synaptic membrane proteins, the detailed subtype localization of 89 differential membrane proteins was further investigated through an integrated database called SynaptomeDB (Table 2) (Pirooznia et al., 2012). The SynaptomeDB compiled the synaptic protein list from a review of all peer-reviewed proteomic studies and from publicly available databases that included proteins in the post- and presynapse, the presynaptic active zone, and the synaptic vesicle, and it provided a detailed and experimentally verified annotation of all known synaptic proteins. Of these detected differential membrane proteins, 81 (91%) were identified by the SynaptomeDB search as having a predicted synapse-specific localization and are the focus of this study (see supplementary Table 3). Figure 6b shows that 41, 31, and 80 proteins were compartmentalized to the presynapse, presynaptic active zone, and postsynaptic density, respectively, and only 6 proteins belong to the synaptic vesicle. A 4-way Venn diagram approach was used to display the overlap between these categories, and multiple overlapping sets were found. Not surprisingly, there were 23 overlapping proteins between the presynapse and presynaptic active zone. On the other hand, some presynaptic proteins, and particularly the active zone associated proteins, were simultaneously found in the postsynaptic density, suggesting that they remained associated with postsynaptic density. This phenomenon is probably due to the “stickiness” of the biochemically isolated fractions and multiple localization of the synaptic proteins.

**Table 2.** Differential Membrane Proteins Identified from the Hippocampal Synaptic Junctions of Susceptible and Unsusceptible Rats

Uniprot Accession	Protein Name	Gene Name	Unique Peptide		Average Fold Change		Function
			Set1	Set2	Susceptible	Unsusceptible	
P61765	Syntaxin-binding protein 1	Stxbp1	69	64	―	1.43	Membrane trafficking
P09951	Synapsin-1	Syn1	97	89	―	1.86	Membrane trafficking
G3V733	Synapsin II, isoform CRA_a	Syn2	41	45	―	1.96	Membrane trafficking
F1M7V4	Protein piccolo	Pclo	54	61	―	1.39	Membrane trafficking
B0BMW0	RAB14, member RAS oncogene family	Rab14	7	6	―	1.34	Membrane trafficking
Q641Z6	EH domain-containing protein 1	Ehd1	21	26	1.39	1.55	Membrane trafficking
Q812E9	Neuronal membrane glycoprotein M6-a	Gpm6a	5	7	2.03	1.58	Membrane trafficking
Q6AXT5	Ras-related protein Rab-21	Rab21	6	7	2.53	―	Membrane trafficking
O54923	Exocyst complex component 6	Exoc6	4	7	―	0.79	Membrane trafficking
P54921	Alpha-soluble NSF attachment protein	Napa	15	21	0.69	0.75	Membrane trafficking
P63012	Ras-related protein Rab-3A	Rab3a	7	8	0.56	0.43	Membrane trafficking
G3V6D3	ATP synthase subunit beta	Atp5b	46	56	0.59	1.76	Transporter
P62815	V-type proton ATPase subunit B, brain isoform	Atp6v1b2	40	35	―	1.57	Transporter
G3V7L8	ATPase, H+ transporting, V1 subunit E isoform 1, isoform CRA_a	Atp6v1e1	20	17	―	1.55	Transporter
D4A133	Protein Atp6v1a	Atp6v1a	41	39	―	1.22	Transporter
P31596	Excitatory amino acid transporter 2	Slc1a2	12	15	0.72	―	Transporter
Q6P6T0	Sideroflexin 3	Sfxn3	4	7	0.60	―	Transporter
P07340	Sodium/potassium-transporting ATPase subunit beta-1	Atp1b1	10	11	0.56	―	Transporter
P15999	ATP synthase subunit alpha, mitochondrial	Atp5a1	39	52	0.70	―	Transporter
P46462	Transitional endoplasmic reticulum ATPase	Vcp	30	30	0.52	―	Transporter
G3V7Q0	Protein Dennd5a	Dennd5a	3	4	―	0.66	Transporter
G3V8Q1	Coatomer protein complex, subunit epsilon (Predicted), isoform CRA_c	Cope	5	7	―	0.65	Transporter
B0BNJ1	LOC683667 protein	Sri	5	4	0.58	0.68	Transporter
Q6P9Y4	ADP/ATP translocase 1	Slc25a4	10	11	0.74	0.52	Transporter
F1LXF1	Protein Bcr (Fragment)	Bcr	13	8	1.42	2.17	Signaling
Q8K3M6	ERC protein 2	Erc2	17	27	―	1.55	Signaling
Q9Z1T4	Connector enhancer of kinase suppressor of ras 2	Cnksr2	10	13	―	1.28	Signaling
Q66HA6	ADP-ribosylation factor-like protein 8B	Arl8b	4	3	―	1.60	Signaling
Q6RUV5	Ras-related C3 botulinum toxin substrate 1	Rac1	11	16	1.53	―	Signaling
P82471	Guanine nucleotide-binding protein G(q) subunit alpha	Gnaq	4	3	1.21	―	Signaling
P11730	Calcium/calmodulin-dependent protein kinase type II subunit gamma	Camk2g	10	12	1.46	―	Signaling
Q6DUV1	Protein kinase C epsilon	Prkce	19	18	0.64	―	Signaling
P61983	14-3-3 protein gamma	Ywhag	10	9	0.62	―	Signaling
P13233	2’,3’-cyclic-nucleotide 3’-phosphodiesterase	Cnp	31	29	0.77	―	Signaling
B5DFC4	Protein kinase C	Prkca	10	6	―	0.60	Signaling
Q9QXK0	Signal transducer and activator of transcription 1	Stat1	4	4	―	0.70	Signaling
P47942	Dihydropyrimidinase-related protein 2	Dpysl2	24	27	0.59	0.71	Signaling
P62260	14-3-3 protein epsilon	Ywhae	22	21	0.49	0.72	Signaling
P63102	14-3-3 protein zeta/delta	Ywhaz	12	14	0.51	0.65	Signaling
Q8VIN2	Annexin	Anxa7	6	7	1.76	1.99	Regulatory/Chaperone
Q5U355	Itfg1 protein	Itfg1	5	3	2.11	1.39	Regulatory/Chaperone
O35274	Neurabin-2	Ppp1r9b	22	23	1.46	―	Regulatory/Chaperone
B0K020	CDGSH iron-sulfur domain- containing protein 1	Cisd1	5	7	1.47	―	Regulatory/Chaperone
Q05175	Brain acid soluble protein 1	Basp1	16	13	0.69	―	Regulatory/Chaperone
F1LP80	Neurosecretory protein VGF	Vgf	5	4	0.50	―	Regulatory/Chaperone
P14669	Annexin A3	Anxa3	18	15	0.53	―	Regulatory/Chaperone
O35095	Neurochondrin	Ncdn	15	14	0.72	―	Regulatory/Chaperone
O35796	Complement component 1 Q subcomponent-binding protein, mitochondrial	C1qbp	8	8	0.74	―	Regulatory/Chaperone
Q66HD0	Endoplasmin	Hsp90b1	21	16	0.55	―	Regulatory/Chaperone
Q6P502	T-complex protein 1 subunit gamma	Cct3	23	21	0.73	―	Regulatory/Chaperone
Q9ERS3	Voltage-dependent calcium channel subunit alpha-2/delta-1	Cacna2d1	31	29	1.50	1.98	Receptor/Channel
O88871	Gamma-aminobutyric acid type B receptor subunit 2	Gabbr2	14	8	1.68	―	Receptor/Channel
Q9Z2L0	Voltage-dependent anion- selective channel protein 1	Vdac1	49	54	1.45	―	Receptor/Channel
D4A3H5	Protein Clcn6	Clcn6	10	10	1.38	―	Receptor/Channel
Q63622	Disks large homolog 2	Dlg2	36	30	1.36	1.60	Scaffolding/Clustering
Q62765	Neuroligin-1	Nlgn1	5	3	―	1.51	Scaffolding/Clustering
Q8R490	Cadherin 13	Cdh13	13	11	―	1.51	Cell adhesion
Q9Z2S9	Flotillin-2	Flot2	21	20	―	1.53	Cell adhesion
D4A8Y0	Protein Cldn12	Cldn12	3	3	―	1.25	Cell adhesion
D4A435	Protein Icam5	Icam5	27	27	0.66	0.54	Cell adhesion
P30427	Plectin	Plec	149	145	2.04	2.01	Cytoskeletal
F1LSL8	Protein Sptbn4	Sptbn4	56	43	1.56	1.75	Cytoskeletal
A2VCW8	Septin 7	Sept7	33	29	―	1.46	Cytoskeletal
Q07266	Drebrin	Dbn1	23	18	1.55	―	Cytoskeletal
F1LSW1	Unconventional myosin-Ib	Myo1b	5	5	1.26	―	Cytoskeletal
Q91ZN1	Coronin-1A	Coro1a	10	7	1.36	―	Cytoskeletal
Q561S0	NADH dehydrogenase [ubiquinone] 1 alpha subcomplex subunit 10, mitochondrial	Ndufa10	17	12	2.37	5.78	Mitochondrial
P04636	Malate dehydrogenase, mitochondrial	Mdh2	17	17	1.38	2.15	Mitochondrial
Q68FY0	Cytochrome b-c1 complex subunit 1, mitochondrial	Uqcrc1	24	20	1.37	1.99	Mitochondrial
P20788	Cytochrome b-c1 complex subunit Rieske, mitochondrial	Uqcrfs1	7	10	―	2.40	Mitochondrial
P11240	Cytochrome c oxidase subunit 5A, mitochondrial	Cox5a	15	17	―	1.67	Mitochondrial
B2RYS2	Cytochrome b-c1 complex subunit 7	Uqcrb	7	10	―	1.55	Mitochondrial
P11951	Cytochrome c oxidase subunit 6C-2	Cox6c2	5	6	―	1.61	Mitochondrial
Q6P6R2	Dihydrolipoyl dehydrogenase, mitochondrial	Dld	33	30	―	1.41	Mitochondrial
Q5XI78	2-oxoglutarate dehydrogenase, mitochondrial	Ogdh	48	49	0.65	―	Mitochondrial
P10860	Glutamate dehydrogenase 1, mitochondrial	Glud1	36	45	0.57	―	Mitochondrial
P08461	Dihydrolipoyllysine-residue acetyltransferase component of pyruvate dehydrogenase complex, mitochondrial	Dlat	23	19	0.55	0.64	Mitochondrial
P49432	Pyruvate dehydrogenase E1 component subunit beta, mitochondrial	Pdhb	19	19	0.53	0.54	Mitochondrial
Q5HZW3	Aspartate beta-hydroxylase domain-containing protein 2	Asphd2	4	3	1.60	2.25	Metabolic
G3V9W6	Aldehyde dehydrogenase	Aldh3a2	4	6	1.65	1.36	Metabolic
Q5XI31	GPI transamidase component PIG-S	Pigs	4	3	1.23	―	Metabolic
Q66HL0	5′ nucleotidase, ecto	Nt5e	8	8	1.47	―	Metabolic
D3ZPU3	Estradiol 17-beta-dehydrogenase 12	Hsd17b12	8	5	1.57	―	Metabolic
B5DEH2	Erlin-2	Erlin2	7	5	1.33	―	Metabolic
P07335	Creatine kinase B-type	Ckb	15	17	0.35	―	Metabolic
P04797	Glyceraldehyde-3-phosphate dehydrogenase	Gapdh	36	41	0.51	―	Metabolic
P12785	Fatty acid synthase	Fasn	50	44	0.71	0.67	Metabolic
Q4FZZ4	Pyruvate dehydrogenase (Lipoamide) alpha 1	Pdha1	19	17	0.58	0.64	Metabolic
I7FKL4	Myelin basic protein transcript variant 1	Mbp	22	21	―	1.46	Other

―, not significantly changed.

### Functional Classification of Differential Membrane Proteins

**Figure 7.**
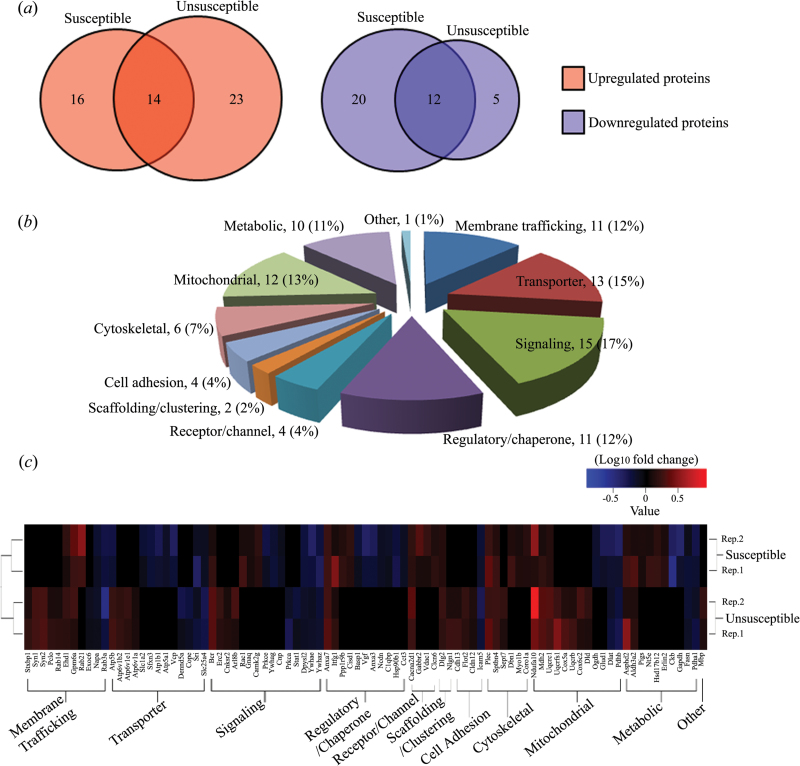
Differentially expressed membrane proteins from hippocampal synaptic junctions. (*a*) Venn diagrams showing the number of uniquely regulated proteins in the susceptible and unsusceptible groups as compared to controls, with the overlap depicting proteins that were identically regulated under both conditions. Upregulated (red) and downregulated (blue) proteins are shown separately. (*b*) Pie chart showing classification of these differentially expressed membrane proteins identified from susceptible and unsusceptible groups. The number of membrane proteins in each category is indicated. (*c*) Upregulated and downregulated proteins are indicated by red and blue color codes, respectively, with the color intensity signifying the expression level as noted in the key bar (top right). Histograms denote the expression trend of the representative proteins.

Here, we designed a quantitative proteomic experiment to explore global patterns of synaptic protein expression in the hippocampus of control, susceptible, and unsusceptible rats. Our goal was to describe 2 main categories of proteins: (1) proteins regulated similarly in susceptible and unsusceptible groups (as a result of exposure to CMS) and (2) proteins regulated differentially in susceptible and unsusceptible rats (which may mediate differences in behavior). Our results, summarized as Venn diagrams in Figure 7a, revealed that the resilience phenotype was much more closely associated with the upregulation of protein groups as a potentially adaptive response. Despite similar abundance changes between the 2 groups, there were a significant number of proteins that displayed changes in abundance levels that were specific to either susceptible or unsusceptible rats, suggesting differences in the molecular mechanism(s) underlying these 2 phenotypes. Furthermore, only one protein, Atp5b, showed an opposite expression trend after CMS application. That is, compared with the control group, the level of Atp5b was significantly upregulated in the unsusceptible group but was downregulated in the susceptible group.

For functional characterization of the differential membrane proteins, their molecular functions were categorized on the basis of their GO functional annotations and literature surveys (Ashburner et al., 2000), though this classification is not strict due to the multiple functions of each protein (Zhou et al., 2010). The identified proteins were classified into 11 functional categories (Figure 7b). From the figure, proteins were involved in extensive synaptic functions, including membrane trafficking, transporter, signaling, regulatory/chaperone, receptor/channel, scaffolding/clustering, cell adhesion, cytoskeletal, mitochondria, metabolic, and other functions. We observed that up to 28% of these differential membrane proteins participated in membrane trafficking and vesicle-mediated transport based on the biological process GO analysis. These proteins have been found to be involved in exocytosis and endocytosis, synaptic vesicle transport and docking, and regulation of the neurotransmitter cycle (Richmond and Broadie, 2002; Schweizer and Ryan, 2006).

To further investigate the relationship between functional clusters and expressional alterations, the dysregulated phenotypes of these differential membrane proteins in each category were systematically analyzed by heatmapping. Figure 7c displays a summary of altered proteins in each functional category, which emphasizes the unique dysregulation of protein expression in the hippocampal synaptic junctions of susceptible and unsusceptible groups. A larger group of proteins involved in membrane trafficking and mitochondrial functions were markedly upregulated in the unsusceptible group, implying their association with CMS resistance. Similarly, we also found that a larger group of proteins involved in regulatory/chaperone activity were significantly changed in susceptible rats. In view of specific alteration patterns of the presynaptic trafficking proteins in the unsusceptible group, we focused on this protein class in the following analysis (Table 2).

### Western-Blot Detection of Stress-Related Presynaptic Trafficking and SNARE Proteins

**Figure 8.**
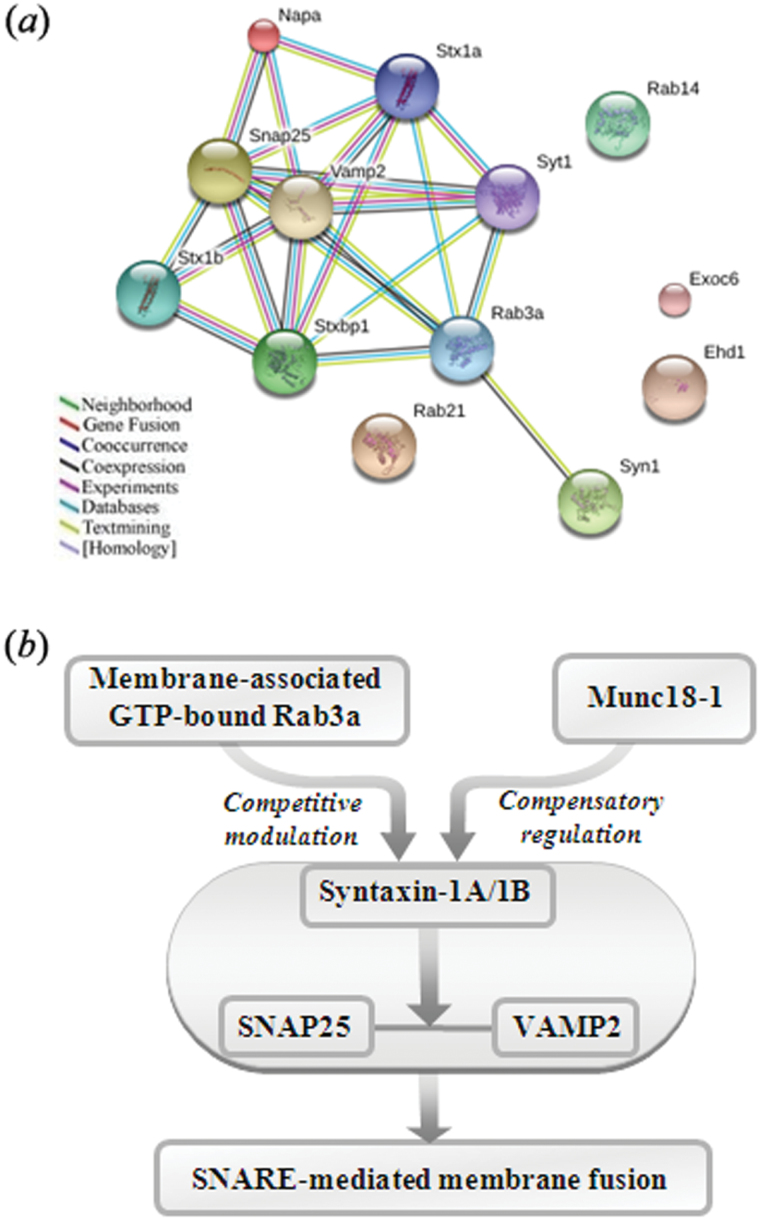
Analysis of synaptic junction proteins involved in membrane trafficking after CMS. (*a*) STRING interaction network of the focus trafficking proteins. Four additional interplay proteins were added to this network. An interaction map was generated using default settings (ie, a high confidence level of 0.7 and 7 linkage criteria: neighborhood, gene fusion, cooccurrence, coexpression, experimental evidence, existing database(s), and text mining). (*b*) Membrane-associated GTP-bound Rab3a and Munc18-1, through co-regulating syntaxin-1/SNAP25/VAMP2 assembly, appears to facilitate soluble N-ethylmaleimide-sensitive factor attachment protein receptor (SNARE)-mediated membrane fusion and resulting neurotransmitter release in the hippocampal presynaptic active zones of unsusceptible rats.

To validate the identification of CMS-responsive proteins detected from the proteomic experiment and to compare the synaptic transmission mechanisms of susceptible and unsusceptible rats, a few membrane proteins of interest (Syn1, Stxbp1 [also known as Munc18-1], and Rab3a) were selected for further analysis by immunoblotting based on in-depth network exploitation of the identified trafficking proteins (Figure 8a) and the availability of commercial antibodies. To probe into the possible mechanism(s), an additional set of 5 targeted proteins involved in presynaptic neurotransmission (ie, the core components of soluble N-ethylmaleimide-sensitive factor attachment protein receptor [SNARE] complex, including Stx1a, Stx1b, SNAP25, VAMP2, and its key regulator Syt1) were also taken into consideration for immunoblotting analysis, even though they were not differentially expressed in the iTRAQ experiments.

**Figure 9.**
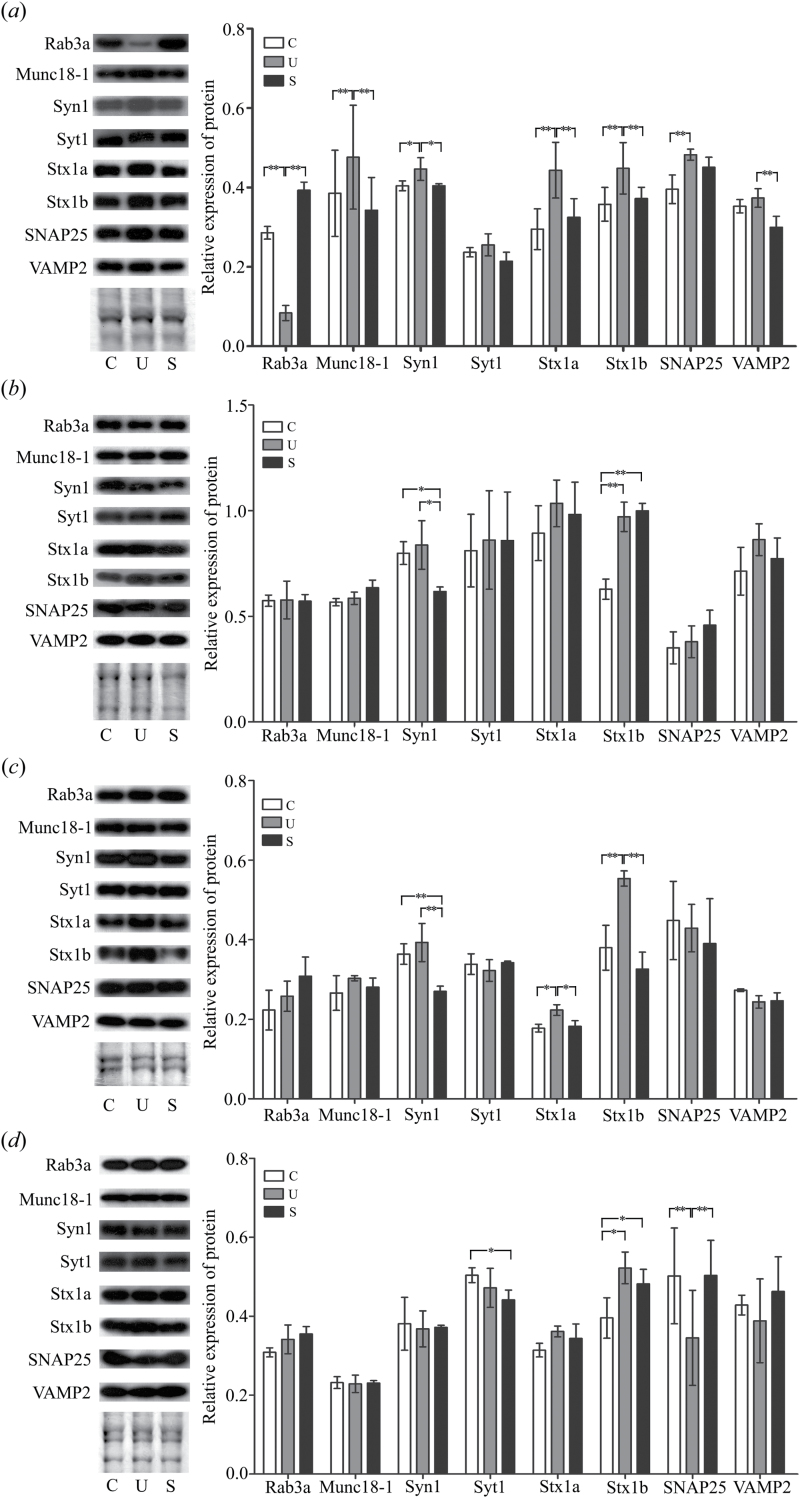
Immunoblotting of select presynaptic membrane proteins. Immunoblotting of the (*a*) synaptic junction, (*b*) Ves, (*c*) Syn, and (*d*) total Hom preparations from hippocampi of the control, susceptible, and unsusceptible groups. Rab3a, Stxbp1 (also known as Munc18-1), Syn1, Syt1, Stx1a, Stx1b, SNAP25, and VAMP2 were detected with their respective antibodies (left). Each blot is representative of triplicate findings, and the protein load was checked by Coomassie-stained gels. The bands for the same proteins were analyzed by densitometry using Quality One software (right). The *X* axis shows the relative intensity. All data were derived from three independent experiments and are shown as mean±SE. *P<.05, **P<.01. C, control; U, unsusceptible; S, susceptible.

As shown in the Western blots (Figure 9a), the expression level of Munc18-1 and Syn1 appeared to be upregulated, whereas Rab3a was significantly downregulated, in synaptic junctional extracts from the unsusceptible group compared with the susceptible and control groups. Moreover, 4 proteins (Stx1a, Stx1b, SNAP25, and VAMP2) of the remaining 5 proteins displayed marked dysregulation by immunoblotting, yet these proteins did not show comparable group regulation patterns in our iTRAQ analysis. Similar discrepancies have also been observed in other proteomic studies (Abdi et al., 2006; Airoldi et al., 2009; Kang et al., 2010; Cheng et al., 2011), probably due to either differences of dynamic range between iTRAQ and immunoblotting (Abdi et al., 2006) or the intrinsic variability associated with the procedural steps of proteomic and immunoblotting analysis. A few potential changes could be masked and missed, which can be partially attributed to the fact that iTRAQ suffers to some extent from the compression of the quantitation ratios to a ratio of 1.0 when used with complex samples. In most cases, changes in the levels as assessed by immunoblotting were larger than observed by iTRAQ; thus, immunoblotting could indicate some results that were not observed in iTRAQ analysis (Dahlhaus et al., 2011).

To gain a more comprehensive view of the data, we examined the different expression patterns of the 8 proteins in the additional subcellular compartmentalization, including the aforementioned Ves, Syn, and Hom preparations (Figure 9b-d). Looking at the whole figure, more alterations of these proteins appeared at synaptic junctions vs the other 3 neuronal fractions. With regard to total Hom extracts (Figure 9d), the decreased expression level of SNAP25 was found in the unsusceptible group when compared with the control and susceptible groups, whereas an opposite trend was shown at the synaptic junctions; this can be partly explained in terms of its known extra-synaptic localization (von Kriegstein and Schmitz, 2003; Hagiwara et al., 2005). Besides Stx1b and Syt1, we found no significant changes in the levels of the other 5 proteins examined in total hippocampal Hom. From the Syn fraction (Figure 9c), the levels of both Stx1a and Stx1b were found to be significantly upregulated in the unsusceptible group compared with both the control and susceptible groups; the same expression pattern was detected in the synaptic junctions. In combination with the results from Figure 9b, the specific CMS insusceptibility-related alterations of Stx1a and Stx1b were found to be nonexistent in the soluble extra-junctional Ves fraction but were found in the insoluble junctional lattices that represent different subcellular compartments (von Kriegstein and Schmitz, 2003; Ribrault et al., 2011). Furthermore, the alterations of Rab3a and Munc18-1 expression levels existed only in the synaptic junctions, which may be concealed by the total protein levels (Maienschein et al., 1999; von Kriegstein and Schmitz, 2003; Yu et al., 2013). Taken together, the distinct phenotypic states of these proteins in multiple compartments of neurons suggest that they interact with several distinct scaffolding proteins and play diverse roles in synapses. In this respect, the synapse-specific alterations of these proteins may be partially covered due to their extra-synaptic expression characteristics (von Kriegstein and Schmitz, 2003; Hagiwara et al., 2005; Ribrault et al., 2011; Yu et al., 2013).

## Discussion

### Segregation of CMS Rats into Susceptible and Unsusceptible Rat Subpopulations

Upon exposure to psychological stress, some individuals are prone to developing mood disorders, whereas others progress normally (Rowland, 2011; Franklin et al., 2012). It has become widely accepted that resilience is not merely a lack of stress susceptibility but is an active process that involves physiological as well as psychological adaptations (Krishnan et al., 2007). Recently, a resilience phenotype has been described in studies employing the chronic social defeat model of depression (Krishnan et al., 2007; Friedman et al., 2014), and stress susceptibility/resilience has also been assessed following exposure to CMS for the importance of this subgroup (Henningsen et al., 2012). Likewise, here we utilized the CMS paradigm to induce diminished responsiveness to a pleasant event, which mimics anhedonia, one of the core symptoms of MDD (Moreau, 2002). This CMS-induced anhedonic-like behavior was measured as a gradual reduction in sucrose preference. Some animals did not reduce their sucrose intake and appeared resilient to developing anhedonic-like behavior in response to stress. A unique feature of this model was that rats exposed to CMS could be segregated into susceptible and unsusceptible populations based on hedonic readouts from sucrose measurements. This segregation was further confirmed by other behavioral findings; anhedonia in susceptible rats was accompanied by increased FST immobility. However, decreased exploratory behavior was found in both susceptible and unsusceptible rats, indicating that these behavioral features were the consequences of CMS independent of anhedonia. In accordance with previous findings (Lucca et al., 2009; Gersner et al., 2010), the locomotor activity measured here was not affected by CMS exposure.

In general, CMS simulates realistic conditions for human depression (Orsetti et al., 2008) and generates multiple behavioral changes similar to those observed clinically, thus supporting the hypothesis that molecular alterations found using this model also occur in human patients with stress-induced depression (Hill et al., 2012). In this study, a separation into susceptible and unsusceptible rats provided a useful approach to identifying molecular factors underlying the mechanisms of stress vulnerability as well as molecular adaptations that promote resistance to stress and adversity. As stress resiliency is a common clinical phenomenon, inclusion of the unsusceptible group raises this model’s value and provides valuable information for resilience-related translational research (Southwick et al., 2005).

### The Hippocampal Synaptic Junction as a Key Substrate for CMS Resistance

Our behavioral findings indicate that, compared with the control and unsusceptible groups, the susceptible group exhibited a markedly reduced sucrose preference and time spent in the central sector of the OFT, and increased immobility time during the FST. However, the unsusceptible group displayed a prominent lack of behavioral phenotypic changes, showing only a decrease of rearing number as compared with controls. In the absence of candidate “resistance-associated proteins,” we designed a quantitative proteomic experiment to explore the effects of CMS on the hippocampal synaptic proteome and to examine the functional consequences of these alterations with particular emphasis on their relevance to stress sensitivity.

Dysregulated synaptic activity is evident in a host of neurological and psychiatric diseases (Jay et al., 2004) such as depression (Duric et al., 2013) and schizophrenia (Zhou et al., 2012b). By conducting synapto-proteomic analysis of CMS depressive rats, we previously revealed some hippocampal synaptic exo-/endocytosis-associated proteins that play various roles in synaptic transmission and plasticity that may underlie the pathoetiology of MDD (Hu et al., 2013). However, it still remains unclear which (if any) distinct events directed by synaptic proteins occurring in the important subregions, particularly at the active zone, correlate with an animal’s response to CMS.

It has been reported that there are synaptic active zones in the synaptic plasma membrane that have critical roles in the release of the neurotransmitter from nerve terminals (Morciano et al., 2009; Sudhof, 2012). The active zone-associated, electron-dense structures (which correspond to synaptic junctions contained in the pre- to postsynaptic scaffold and presynaptic web) were biochemically characterized by the insolubility in TX-100 (Phillips et al., 2001; Li et al., 2009). For this study, a pH 6 solution containing 1% TX-100 was used to dissolve some molecules that were primarily associated with synaptic vesicles as well as molecules loosely connected to the synaptic junctional scaffold. As expected, the molecules involved in synaptic vesicle dynamics at the presynaptic membrane would be retained in the detergent-insoluble pH 6 synaptic junctional pellet if they were connected to the synaptic scaffold. Thus, in agreement with previously published studies, the synaptosomal- and synaptic junction-enriched fractionation protocol described herein yielded an enrichment of proteins known to be localized to the synaptic active zone with a reduction of cytomatrix proteins. Some presynaptic active zone proteins (such as syntaxin-1, SNAP25, and Munc18-1) were present in the insoluble junctional fraction, which is consistent with the localization of these proteins within the presynaptic membrane specialization and possibly within the 50-nm presynaptic particles themselves (Sudhof, 2012). A significant proportion of the proteins was solubilized; however, Figure 9 revealed synaptic and nonsynaptic pools of these molecules (von Kriegstein and Schmitz, 2003; Hagiwara et al., 2005; Ribrault et al., 2011; Yu et al., 2013), and the TX-100 (pH 6) did not solubilize the junction-associated pools of these presynaptic proteins. In fact, many studies clearly showed that the presynaptic web forms a subset of interrelated proteins embedded in the presynaptic membrane specialization, which is composed of synaptic vesicle exocytosis and recycled proteins.

In our study, by means of the biochemical fractionation approach, the enriched synaptic junctions as a molecular vessel served to intensively investigate the complexity of molecular changes hidden at the synaptic active zone through the following iTRAQ-based proteomic analysis. Neurotransmission, synaptic contact, and the short- and long-term structural and functional dynamics are governed by a unique set of proteins at the active zone. The identification of the protein inventory in the synaptic active zone is of utmost importance in understanding the regulation and modulation of chemical signaling along with its pathologies.

To obtain information about the 2 stress-sensitivity phenotypes, the hippocampal synaptic protein profiles from the 3 experimental groups (control, susceptible, and unsusceptible) were established and compared. iTRAQ-labeled peptides were separated and identified by LC-MS/MS, which provided a sensitive and robust quantitative proteomic platform for identifying synaptic molecules of stress reactivity. Functional cluster analysis on all synaptic membrane proteins indicated that, in terms of protein expression levels in the hippocampal synaptic active zone, the unsusceptible phenotype had a distinctly different proteomic profile with respect to protein systems involved in membrane trafficking, transporter, regulatory/chaperone, and mitochondrial functions (Figure 7c). These differential expressions of various proteins indicate that the hippocampal synaptic active zone might be the vital substrate involved with resistance to CMS. Importantly, the unbiased profiling results from this study revealed a series of candidate synaptic proteins whose alterations occurred in the nerve terminal active zone of the unsusceptible group, suggesting that the expression of this phenotype is an active neurobiological process that is not simply the absence of vulnerability (Krishnan et al., 2007).

### Rab3a and Munc18-1 Coregulation as a Potential Molecular Adaptation Facilitating SNARE-Mediated Membrane Fusion

An interesting finding was that the more significantly upregulated proteins in a specific profile for unsusceptible rats, as compared with both the control and susceptible rats, were found to be closely associated with membrane trafficking in synaptic transmission. It can be surmised that expressional changes in these proteins may be involved in the molecular adaptation and stress-coping mechanism in unsusceptible rats. To our knowledge, the presynaptic active zone, characterized by numerous synaptic vesicles docked to the presynaptic plasma membrane (Lin and Scheller, 2000; Owald and Sigrist, 2009), is the key site of synaptic vesicle fusion and is thus of central importance for the chemical communication between neurons and neighboring cells (Owald and Sigrist, 2009; Sudhof, 2012). The docking process of synaptic vesicles is mediated by the concerted action of 3 proteins (syntaxin-1, SNAP25, and VAMP2) that form a highly stable bundle of 4 parallel α-helices (the core SNARE complex) that drives vesicle fusion (Rothman, 1994; Jahn and Sudhof, 1999; Snyder et al., 2006). Previous studies have indicated that SNARE protein expression levels affect docking, priming, and release probabilities (Ramakrishnan et al., 2012), and the assembly process of syntaxin-1/SNAP25/VAMP2 in the presynaptic active zone controls presynaptic neurotransmitter release (Brunger, 2000; Owald and Sigrist, 2009).

Centered around this core machinery for membrane fusion, we combined all the identified differential trafficking proteins with the core SNARE components to create a network map through STRING analysis in an attempt to find possible internal linkages and associated partners. As shown in Figure 8a, 4 proteins from the aforementioned proteomic analysis, namely Rab3a, Munc18-1, Syn1, and Napa, were highly correlated with SNARE-mediated membrane fusion.

Through further selective immunoblotting of the 4 different fractions, we found an accumulation of membrane-associated Rab3a form in the unsusceptible group as compared with the control and susceptible groups; this protein is GTP-bound (not cytosolic GDP-bound) and is therefore the active form (Gawinecka et al., 2012; Chen et al., 2013). Rab3a is a small neuronal GTP-binding protein that localizes to synaptic vesicles and plays a key regulatory role in Ca^2+^-dependent exocytosis, particularly in neurotransmitter release from nerve terminals (Sudhof, 2004; Sakane et al., 2006). Rab3a has a function upstream of vesicle fusion in the activity-dependent transport of synaptic vesicles to and from their docking in the active zone (Leenders et al., 2001). As a GTP-dependent molecular switch, Rab3a is able to improve the fidelity of protein-protein interactions at the targets of a transport step, such as the pairing of SNARE proteins (Sogaard et al., 1994; Novick and Zerial, 1997; Schimmoller et al., 1998; Gonzalez and Scheller, 1999) and acting upstream of SNARE complex formation between the vesicle and target membrane (Geppert et al., 1994; Johannes et al., 1996; Geppert et al., 1997; Silinsky, 2008; Degtyar et al., 2013). Some previous studies have found that Rab3a is an interacting partner of Syt1 and may participate in the regulation of synaptic membrane fusion by competitively modulating the interaction of synaptotagmin with syntaxin-1 of the SNARE complex in presynaptic membranes (Horikawa et al., 1993; Xie et al., 2014). Intriguingly, in the presynaptic active zone, we found elevated levels of syntaxin-1A/1B in the unsusceptible phenotype relative to the control and susceptible groups, possibly due to direct competition between the membrane-associated GTP-bound Rab3a and syntaxin-1 for the same binding site within the C2B domain of Syt1 (Xie et al., 2014). Moreover, the expression of Syt1 (as an exocytic Ca^2+^ sensor) showed no significant change in the active zone of the synapse (Kerr et al., 2008). Therefore, we speculate that the large reduction of GTP-bound Rab3a at the synaptic active zone of unsusceptible rats may lead to its reduced interaction with Syt1, which in turn leads to an increased interaction of Syt1 with syntaxin-1, thereby promoting SNARE complex formation and facilitating membrane fusion during exocytosis (Takai et al., 1996; Xie et al., 2014).

Syntaxin-1 and Munc18-1 have been postulated to function as docking and fusion platforms for synaptic vesicles (Voets et al., 2001; Gulyas-Kovacs et al., 2007; Camoletto et al., 2009). Munc18-1 is a molecular chaperone of syntaxin-1 by virtue of its tight binding (Graham et al., 2004; Hu et al., 2011), which is involved in SNARE-mediated membrane fusion and the docking of large dense-core vesicles to the plasma membrane (Toonen, 2003; Han et al., 2010). In this study, Munc18-1 expression was found to be upregulated in the active zone of unsusceptible rats, thereby directly promoting syntaxin-1 stability and regulating the formation of vesicle priming. Thus, elevated Munc18-1 may act as a compensatory regulator of accelerated syntaxin-1 (Zilly et al., 2006), binding simultaneously to the SNARE complex to control the assembly of the Munc18-1/SNARE membrane fusion complex (Togneri et al., 2006; Zilly et al., 2006; Khvotchev et al., 2007; Rathore et al., 2010; Lim et al., 2013).

Finally, to address whether additional core components of the SNARE complex were affected, SNAP25 and VAMP2 expression in the synaptic junction was analyzed. SNAP-25 is a membrane-bound protein anchored via the palmitoylation of cysteines in the linker region that connects the 2 SNARE motifs (Fukuda et al., 2000). Syntaxin-1 and VAMP2 are anchored via transmembrane domains (Degtyar et al., 2013). After the docking and priming of synaptic vesicles, VAMP2 interacts with SNAP-25 and syntaxin-1 to form a transient SNARE complex that mediates membrane fusion by bringing the vesicle and the presynaptic plasma membrane at the active zone to release the neurotransmitter (Sudhof and Rothman, 2009). In unsusceptible rats, significant SNAP25 upregulation was observed compared with controls, and VAMP2 also showed a similar upregulation when compared with susceptible rats. These findings reveal that the syntaxin-1/SNAP25/VAMP2 assembly (Otto et al., 1997) for the fusion event was affected in the unsusceptible rats. This effect shows a high level of specificity for stress insusceptibility that can be demonstrated by particular changes in the relative amounts of SNARE proteins.

In sum, our findings support a molecular adaptation on synaptic transmission in unsusceptible rats and especially on SNARE-related components. The accessory proteins (membrane-associated GTP-bound Rab3a and Munc18-1) may facilitate SNARE-mediated membrane fusion and the subsequent release and recycling of neurotransmitters by coregulating syntaxin-1/SNAP25/VAMP2 assembly at the presynaptic active zone of unsusceptible rats (Figure 8b) (Bock and Scheller, 1999; Chen and Scheller, 2001). However, the precise mechanism(s) that orchestrate the differential behavioral responses to stress, with particular respect to stress resilience, still require further investigation.

## Conclusions

In this study, we used a quantitative proteomic approach to investigate the alterations of synaptic junctional protein expression in the hippocampus of rats subjected to CMS. The unbiased profiles identified several candidate proteins in the synaptic active zone that may be related to stress vulnerability or insusceptibility and provide insight into the pathogenesis of stress-related disorders. On the subcellular proteome level, this study provides preliminary evidence that protein modulations in the synaptic active zone are causally linked to behavioral adaptations to stress. Moreover, our data support the concept that there is dysregulation of synaptic transmission and protein systems particularly involved in membrane trafficking in the active zone of unsusceptible rats, revealing new investigative protein targets that may contribute to a better understanding of stress resilience. Through STRING and immunoblotting analysis, membrane-associated GTP-bound Rab3a and Munc18-1 appear to coregulate syntaxin-1/SNAP25/VAMP2 assembly at the hippocampal presynaptic active zone of unsusceptible rats, thereby facilitating SNARE-mediated membrane fusion and neurotransmitter release. The activity of these 2 proteins may be a part of a stress-protection mechanism that actively maintains emotional homeostasis under stressful conditions.

## Interest Statement

None.

## Supplementary Material

supplementary Methods

## References

[CIT0001] Abdi F (2006) Detection of biomarkers with a multiplex quantitative proteomic platform in cerebrospinal fluid of patients with neurodegenerative disorders. J Alzheimers Dis 9:293–348. 16914840 10.3233/jad-2006-9309

[CIT0002] Abul-Husn NS Bushlin I Moron JA Jenkins SL Dolios G Wang R Iyengar R Ma’ayan A Devi LA (2009) Systems approach to explore components and interactions in the presynapse. Proteomics 9:3303–3315. 19562802 10.1002/pmic.200800767 PMC2766278

[CIT0003] Airoldi L Magagnotti C Iannuzzi AR Marelli C Bagnati R Pastorelli R Colombi A Santaguida S Chiabrando C Schiarea S Fanelli R (2009) Effects of cigarette smoking on the human urinary proteome. Biochem Biophys Res Commun 381:397–402. 19222993 10.1016/j.bbrc.2009.02.055

[CIT0004] Ashburner M Ball CA Blake JA Botstein D Butler H Cherry JM Davis AP Dolinski K Dwight SS Eppig JT Harris MA Hill DP Issel-Tarver L Kasarskis A Lewis S Matese JC Richardson JE Ringwald M Rubin GM Sherlock G (2000) Gene ontology: tool for the unification of biology. The Gene Ontology Consortium. Nat Genet 25:25–29. 10802651 10.1038/75556 PMC3037419

[CIT0005] Bannerman DM Bus T Taylor A Sanderson DJ Schwarz I Jensen V Hvalby O Rawlins JN Seeburg PH Sprengel R (2012) Dissecting spatial knowledge from spatial choice by hippocampal NMDA receptor deletion. Nat Neurosci 15:1153–1159. 22797694 10.1038/nn.3166 PMC3442238

[CIT0006] Bannerman DM Sprengel R Sanderson DJ McHugh SB Rawlins JN Monyer H Seeburg PH (2014) Hippocampal synaptic plasticity, spatial memory and anxiety. Nat Rev Neurosci 15:181–192. 24552786 10.1038/nrn3677

[CIT0007] Bliss TV Collingridge GL (1993) A synaptic model of memory: long-term potentiation in the hippocampus. Nature 361:31–39. 8421494 10.1038/361031a0

[CIT0008] Bock JB Scheller RH (1999) SNARE proteins mediate lipid bilayer fusion. Proc Natl Acad Sci U S A 96:12227–12229. 10535902 10.1073/pnas.96.22.12227 PMC34255

[CIT0009] Brunger AT (2000) Structural insights into the molecular mechanism of Ca(2+)-dependent exocytosis. Curr Opin Neurobiol 10:293–302. 10851178 10.1016/s0959-4388(00)00098-2

[CIT0010] Camoletto PG Vara H Morando L Connell E Marletto FP Giustetto M Sassoe-Pognetto M Van Veldhoven PP Ledesma MD (2009) Synaptic vesicle docking: sphingosine regulates syntaxin1 interaction with Munc18. PLoS One 4:e5310. 19390577 10.1371/journal.pone.0005310 PMC2668795

[CIT0011] Charney DS (2004) Psychobiological mechanisms of resilience and vulnerability: implications for successful adaptation to extreme stress. Am J Psychiatry 161:195–216. 14754765 10.1176/appi.ajp.161.2.195

[CIT0012] Chen RH Wislet-Gendebien S Samuel F Visanji NP Zhang G Marsilio D Langman T Fraser PE Tandon A (2013) alpha-Synuclein membrane association is regulated by the Rab3a recycling machinery and presynaptic activity. J Biol Chem 288:7438–7449. 23344955 10.1074/jbc.M112.439497 PMC3597785

[CIT0013] Chen YA Scheller RH (2001) SNARE-mediated membrane fusion. Nat Rev Mol Cell Biol 2:98–106. 11252968 10.1038/35052017

[CIT0014] Cheng PJ Wang TH Huang SY Kao CC Lu JH Hsiao CH Shaw SW (2011) Differential proteomics analysis of amniotic fluid in pregnancies of increased nuchal translucency with normal karyotype. Prenat Diagn 31:274–281. 21312199 10.1002/pd.2719

[CIT0015] Christoffel DJ Golden SA Russo SJ (2011) Structural and synaptic plasticity in stress-related disorders. Rev Neurosci 22:535–549. 21967517 10.1515/RNS.2011.044 PMC3212803

[CIT0016] Counotte DS Li KW Wortel J Gouwenberg Y Van Der Schors RC Smit AB Spijker S (2010) Changes in molecular composition of rat medial prefrontal cortex synapses during adolescent development. Eur J Neurosci 32:1452–1460. 20950357 10.1111/j.1460-9568.2010.07404.x

[CIT0017] Dahlhaus M Li KW van der Schors RC Saiepour MH van Nierop P Heimel JA Hermans JM Loos M Smit AB Levelt CN (2011) The synaptic proteome during development and plasticity of the mouse visual cortex. Mol Cell Proteomics 10:M110 005413. 21398567 10.1074/mcp.M110.005413 PMC3098591

[CIT0018] Degtyar V Hafez IM Bray C Zucker RS (2013) Dance of the SNAREs: assembly and rearrangements detected with FRET at neuronal synapses. J Neurosci 33:5507–5523. 23536066 10.1523/JNEUROSCI.2337-12.2013 PMC3647469

[CIT0019] Delgado y Palacios R Campo A Henningsen K Verhoye M Poot D Dijkstra J Van Audekerke J Benveniste H Sijbers J Wiborg O Van der Linden A (2011) Magnetic resonance imaging and spectroscopy reveal differential hippocampal changes in anhedonic and resilient subtypes of the chronic mild stress rat model. Biol Psychiatry 70:449–457. 21762877 10.1016/j.biopsych.2011.05.014

[CIT0020] Duman RS Aghajanian GK (2012) Synaptic dysfunction in depression: potential therapeutic targets. Science 338:68–72. 23042884 10.1126/science.1222939 PMC4424898

[CIT0021] Duric V Banasr M Stockmeier CA Simen AA Newton SS Overholser JC Jurjus GJ Dieter L Duman RS (2013) Altered expression of synapse and glutamate related genes in post-mortem hippocampus of depressed subjects. Int J Neuropsychopharmacol 16:69–82. 22339950 10.1017/S1461145712000016 PMC3414647

[CIT0022] Feder A Nestler EJ Charney DS (2009) Psychobiology and molecular genetics of resilience. Nat Rev Neurosci 10:446–457. 19455174 10.1038/nrn2649 PMC2833107

[CIT0023] Fleshner M Maier SF Lyons DM Raskind MA (2011) The neurobiology of the stress-resistant brain. Stress 14:498–502. 21790482 10.3109/10253890.2011.596865 PMC3287388

[CIT0024] Franklin TB Saab BJ Mansuy IM (2012) Neural mechanisms of stress resilience and vulnerability. Neuron 75:747–761. 22958817 10.1016/j.neuron.2012.08.016

[CIT0025] Friedman AK Walsh JJ Juarez B Ku SM Chaudhury D Wang J Li X Dietz DM Pan N Vialou VF Neve RL Yue Z Han MH (2014) Enhancing depression mechanisms in midbrain dopamine neurons achieves homeostatic resilience. Science 344:313–319. 24744379 10.1126/science.1249240 PMC4334447

[CIT0026] Fukuda R McNew JA Weber T Parlati F Engel T Nickel W Rothman JE Sollner TH (2000) Functional architecture of an intracellular membrane t-SNARE. Nature 407:198–202. 11001059 10.1038/35025084

[CIT0027] Gawinecka J Cardone F Asif AR De Pascalis A Wemheuer WM Schulz-Schaeffer WJ Pocchiari M Zerr I (2012) Sporadic Creutzfeldt-Jakob disease subtype-specific alterations of the brain proteome: impact on Rab3a recycling. Proteomics 12:3610–3620. 23070823 10.1002/pmic.201200201 PMC3565451

[CIT0028] Geppert M Bolshakov VY Siegelbaum SA Takei K De Camilli P Hammer RE Sudhof TC (1994) The role of Rab3A in neurotransmitter release. Nature 369:493–497. 7911226 10.1038/369493a0

[CIT0029] Geppert M Goda Y Stevens CF Sudhof TC (1997) The small GTP-binding protein Rab3A regulates a late step in synaptic vesicle fusion. Nature 387:810–814. 9194562 10.1038/42954

[CIT0030] Gersner R Toth E Isserles M Zangen A (2010) Site-specific antidepressant effects of repeated subconvulsive electrical stimulation: potential role of brain-derived neurotrophic factor. Biol Psychiatry 67:125–132. 19880094 10.1016/j.biopsych.2009.09.015

[CIT0031] Gonzalez L Jr Scheller RH (1999) Regulation of membrane trafficking: structural insights from a Rab/effector complex. Cell 96:755–758. 10102263 10.1016/s0092-8674(00)80585-1

[CIT0032] Gourley SL Swanson AM Koleske AJ (2013) Corticosteroid-induced neural remodeling predicts behavioral vulnerability and resilience. J Neurosci 33:3107–3112. 23407965 10.1523/JNEUROSCI.2138-12.2013 PMC3711631

[CIT0033] Graham ME Barclay JW Burgoyne RD (2004) Syntaxin/Munc18 interactions in the late events during vesicle fusion and release in exocytosis. J Biol Chem 279:32751–32760. 15175344 10.1074/jbc.M400827200

[CIT0034] Grippo AJ Beltz TG Weiss RM Johnson AK (2006) The effects of chronic fluoxetine treatment on chronic mild stress-induced cardiovascular changes and anhedonia. Biol Psychiatry 59:309–316. 16154542 10.1016/j.biopsych.2005.07.010

[CIT0035] Gulyas-Kovacs A de Wit H Milosevic I Kochubey O Toonen R Klingauf J Verhage M Sorensen JB (2007) Munc18-1: sequential interactions with the fusion machinery stimulate vesicle docking and priming. J Neurosci 27:8676–8686. 17687045 10.1523/JNEUROSCI.0658-07.2007 PMC6672934

[CIT0036] Hagiwara A Fukazawa Y Deguchi-Tawarada M Ohtsuka T Shigemoto R (2005) Differential distribution of release-related proteins in the hippocampal CA3 area as revealed by freeze-fracture replica labeling. J Comp Neurol 489:195–216. 15983999 10.1002/cne.20633

[CIT0037] Han GA Malintan NT Collins BM Meunier FA Sugita S (2010) Munc18-1 as a key regulator of neurosecretion. J Neurochem 115:1–10. 20681955 10.1111/j.1471-4159.2010.06900.x

[CIT0038] Henningsen K Palmfeldt J Christiansen S Baiges I Bak S Jensen ON Gregersen N Wiborg O (2012) Candidate hippocampal biomarkers of susceptibility and resilience to stress in a rat model of depression. Mol Cell Proteomics 11:M111 016428. 22311638 10.1074/mcp.M111.016428 PMC3394954

[CIT0039] Hill MN Hellemans KG Verma P Gorzalka BB Weinberg J (2012) Neurobiology of chronic mild stress: parallels to major depression. Neurosci Biobehav Rev 36:2085–2117. 22776763 10.1016/j.neubiorev.2012.07.001 PMC4821201

[CIT0040] Holderbach R Clark K Moreau JL Bischofberger J Normann C (2007) Enhanced long-term synaptic depression in an animal model of depression. Biol Psychiatry 62:92–100. 17141742 10.1016/j.biopsych.2006.07.007

[CIT0041] Horikawa HP Saisu H Ishizuka T Sekine Y Tsugita A Odani S Abe T (1993) A complex of rab3A, SNAP-25, VAMP/synaptobrevin-2 and syntaxins in brain presynaptic terminals. FEBS Lett 330:236–240. 8365494 10.1016/0014-5793(93)80281-x

[CIT0042] Hu SH Christie MP Saez NJ Latham CF Jarrott R Lua LH Collins BM Martin JL (2011) Possible roles for Munc18-1 domain 3a and Syntaxin1 N-peptide and C-terminal anchor in SNARE complex formation. Proc Natl Acad Sci U S A 108:1040–1045. 21193638 10.1073/pnas.0914906108 PMC3024693

[CIT0043] Hu Y Zhou J Fang L Liu H Zhan Q Luo D Zhou C Chen J Li Q Xie P (2013) Hippocampal synaptic dysregulation of exo/endocytosis-associated proteins induced in a chronic mild-stressed rat model. Neuroscience 230:1–12. 22922266 10.1016/j.neuroscience.2012.08.026

[CIT0044] Huang da W Sherman BT Lempicki RA (2009) Systematic and integrative analysis of large gene lists using DAVID bioinformatics resources. Nat Protoc 4:44–57. 19131956 10.1038/nprot.2008.211

[CIT0045] Jahn R Sudhof TC (1999) Membrane fusion and exocytosis. Annu Rev Biochem 68:863–911. 10872468 10.1146/annurev.biochem.68.1.863

[CIT0046] Jay TM Rocher C Hotte M Naudon L Gurden H Spedding M (2004) Plasticity at hippocampal to prefrontal cortex synapses is impaired by loss of dopamine and stress: importance for psychiatric diseases. Neurotox Res 6:233–244. 15325962 10.1007/BF03033225

[CIT0047] Johannes L Doussau F Clabecq A Henry JP Darchen F Poulain B (1996) Evidence for a functional link between Rab3 and the SNARE complex. J Cell Sci 109 (Pt 12):2875–2884. 9013335 10.1242/jcs.109.12.2875

[CIT0048] Kamal A Ramakers GM Altinbilek B Kas MJ (2014) Social isolation stress reduces hippocampal long-term potentiation: effect of animal strain and involvement of glucocorticoid receptors. Neuroscience 256:262–270. 24161282 10.1016/j.neuroscience.2013.10.016

[CIT0049] Kang UB Ahn Y Lee JW Kim YH Kim J Yu MH Noh DY Lee C (2010) Differential profiling of breast cancer plasma proteome by isotope-coded affinity tagging method reveals biotinidase as a breast cancer biomarker. BMC Cancer 10:114. 20346108 10.1186/1471-2407-10-114 PMC2861033

[CIT0050] Kempermann G Kronenberg G (2003) Depressed new neurons--adult hippocampal neurogenesis and a cellular plasticity hypothesis of major depression. Biol Psychiatry 54:499–503. 12946878 10.1016/s0006-3223(03)00319-6

[CIT0051] Kendler KS Karkowski LM Prescott CA (1999) Causal relationship between stressful life events and the onset of major depression. Am J Psychiatry 156:837–841. 10360120 10.1176/ajp.156.6.837

[CIT0052] Kerr AM Reisinger E Jonas P (2008) Differential dependence of phasic transmitter release on synaptotagmin 1 at GABAergic and glutamatergic hippocampal synapses. Proc Natl Acad Sci U S A 105:15581–15586. 18832148 10.1073/pnas.0800621105 PMC2563064

[CIT0053] Kessler RC Berglund P Demler O Jin R Koretz D Merikangas KR Rush AJ Walters EE Wang PS, National Comorbidity Survey R (2003) The epidemiology of major depressive disorder: results from the National Comorbidity Survey Replication (NCS-R). JAMA 289:3095–3105. 12813115 10.1001/jama.289.23.3095

[CIT0054] Khvotchev M Dulubova I Sun J Dai H Rizo J Sudhof TC (2007) Dual modes of Munc18-1/SNARE interactions are coupled by functionally critical binding to syntaxin-1 N terminus. J Neurosci 27:12147–12155. 17989281 10.1523/JNEUROSCI.3655-07.2007 PMC6673268

[CIT0055] Kim JI Lee JW Lee YA Lee DH Han NS Choi YK Hwang BR Kim HJ Han JS (2013) Sexual activity counteracts the suppressive effects of chronic stress on adult hippocampal neurogenesis and recognition memory. Brain Res 1538:26–40. 24041775 10.1016/j.brainres.2013.09.007

[CIT0056] Kim JJ Song EY Kosten TA (2006) Stress effects in the hippocampus: synaptic plasticity and memory. Stress 9:1–11. 16753928 10.1080/10253890600678004

[CIT0057] Krishnan V, Han MH, Graham DL, Berton O, Renthal W, Russo SJ, Laplant Q, Graham A, Lutter M, Lagace DC, Ghose S, Reister R, Tannous P, Green TA, Neve RL, Chakravarty S, Kumar A, Eisch AJ, Self DW, Lee FS et al. (2007) Molecular adaptations underlying susceptibility and resistance to social defeat in brain reward regions. Cell 131:391–404. 17956738 10.1016/j.cell.2007.09.018

[CIT0058] Leenders AG Lopes da Silva FH Ghijsen WE Verhage M (2001) Rab3a is involved in transport of synaptic vesicles to the active zone in mouse brain nerve terminals. Mol Biol Cell 12:3095–3102. 11598194 10.1091/mbc.12.10.3095 PMC60158

[CIT0059] Leuner B Gould E (2010) Structural plasticity and hippocampal function. Annu Rev Psychol 61:111–140, C111–113. 19575621 10.1146/annurev.psych.093008.100359 PMC3012424

[CIT0060] Li X Xie C Jin Q Liu M He Q Cao R Lin Y Li J Li Y Chen P Liang S (2009) Proteomic screen for multiprotein complexes in synaptic plasma membrane from rat hippocampus by blue native gel electrophoresis and tandem mass spectrometry. J Proteome Res 8:3475–3486. 19432478 10.1021/pr900101d

[CIT0061] Lim SH Moon J Lee M Lee JR (2013) PTPRT regulates the interaction of Syntaxin-binding protein 1 with Syntaxin 1 through dephosphorylation of specific tyrosine residue. Biochem Biophys Res Commun 439:40–46. 23962429 10.1016/j.bbrc.2013.08.033

[CIT0062] Lin RC Scheller RH (2000) Mechanisms of synaptic vesicle exocytosis. Annu Rev Cell Dev Biol 16:19–49. 11031229 10.1146/annurev.cellbio.16.1.19

[CIT0063] Lin XQ Liang SL Han SY Zheng SP Ye YR Lin Y (2013) Quantitative iTRAQ LC-MS/MS proteomics reveals the cellular response to heterologous protein overexpression and the regulation of HAC1 in Pichia pastoris. J Proteomics 91:58–72. 23851310 10.1016/j.jprot.2013.06.031

[CIT0064] Liu T Hu J Li H (2009) iTRAQ-based shotgun neuroproteomics. Methods Mol Biol 566:201–216. 20058174 10.1007/978-1-59745-562-6_14 PMC4640694

[CIT0065] Lucca G Comim CM Valvassori SS Reus GZ Vuolo F Petronilho F Gavioli EC Dal-Pizzol F Quevedo J (2009) Increased oxidative stress in submitochondrial particles into the brain of rats submitted to the chronic mild stress paradigm. J Psychiatr Res 43:864–869. 19100996 10.1016/j.jpsychires.2008.11.002

[CIT0066] Maggio N Segal M (2011) Persistent changes in ability to express long-term potentiation/depression in the rat hippocampus after juvenile/adult stress. Biol Psychiatry 69:748–753. 21216393 10.1016/j.biopsych.2010.11.026

[CIT0067] Maienschein V Marxen M Volknandt W Zimmermann H (1999) A plethora of presynaptic proteins associated with ATP-storing organelles in cultured astrocytes. Glia 26:233–244. 10340764 10.1002/(sici)1098-1136(199905)26:3<233::aid-glia5>3.0.co;2-2

[CIT0068] Maras PM Baram TZ (2012) Sculpting the hippocampus from within: stress, spines, and CRH. Trends Neurosci 35:315–324. 22386641 10.1016/j.tins.2012.01.005 PMC3423222

[CIT0069] Marrocco J Mairesse J Ngomba RT Silletti V Van Camp G Bouwalerh H Summa M Pittaluga A Nicoletti F Maccari S Morley-Fletcher S (2012) Anxiety-like behavior of prenatally stressed rats is associated with a selective reduction of glutamate release in the ventral hippocampus. J Neurosci 32:17143–17154. 23197707 10.1523/JNEUROSCI.1040-12.2012 PMC6621858

[CIT0070] McEwen BS (2010) Stress, sex, and neural adaptation to a changing environment: mechanisms of neuronal remodeling. Ann N Y Acad Sci 1204 Suppl:E38–59. 20840167 10.1111/j.1749-6632.2010.05568.x PMC2946089

[CIT0071] McEwen BS Eiland L Hunter RG Miller MM (2012) Stress and anxiety: structural plasticity and epigenetic regulation as a consequence of stress. Neuropharmacology 62:3–12. 21807003 10.1016/j.neuropharm.2011.07.014 PMC3196296

[CIT0072] Morciano M Beckhaus T Karas M Zimmermann H Volknandt W (2009) The proteome of the presynaptic active zone: from docked synaptic vesicles to adhesion molecules and maxi-channels. J Neurochem 108:662–675. 19187093 10.1111/j.1471-4159.2008.05824.x

[CIT0073] Moreau JL (2002) Simulating the anhedonia symptom of depression in animals. Dialogues Clin Neurosci 4:351–360. 22034464 10.31887/DCNS.2002.4.4/jlmoreau PMC3181703

[CIT0074] Muller HK Wegener G Popoli M Elfving B (2011) Differential expression of synaptic proteins after chronic restraint stress in rat prefrontal cortex and hippocampus. Brain Res 1385:26–37. 21354112 10.1016/j.brainres.2011.02.048

[CIT0075] Nestler EJ Barrot M DiLeone RJ Eisch AJ Gold SJ Monteggia LM (2002) Neurobiology of depression. Neuron 34:13–25. 11931738 10.1016/s0896-6273(02)00653-0

[CIT0076] Novick P Zerial M (1997) The diversity of Rab proteins in vesicle transport. Curr Opin Cell Biol 9:496–504. 9261061 10.1016/s0955-0674(97)80025-7

[CIT0077] Orsetti M Di Brisco F Canonico PL Genazzani AA Ghi P (2008) Gene regulation in the frontal cortex of rats exposed to the chronic mild stress paradigm, an animal model of human depression. Eur J Neurosci 27:2156–2164. 18371075 10.1111/j.1460-9568.2008.06155.x

[CIT0078] Otto H Hanson PI Jahn R (1997) Assembly and disassembly of a ternary complex of synaptobrevin, syntaxin, and SNAP-25 in the membrane of synaptic vesicles. Proc Natl Acad Sci U S A 94:6197–6201. 9177194 10.1073/pnas.94.12.6197 PMC21026

[CIT0079] Owald D Sigrist SJ (2009) Assembling the presynaptic active zone. Curr Opin Neurobiol 19:311–318. 19395253 10.1016/j.conb.2009.03.003

[CIT0080] Paizanis E Hamon M Lanfumey L (2007) Hippocampal neurogenesis, depressive disorders, and antidepressant therapy. Neural Plast 2007:73754. 17641737 10.1155/2007/73754 PMC1906869

[CIT0081] Phillips GR Huang JK Wang Y Tanaka H Shapiro L Zhang W Shan WS Arndt K Frank M Gordon RE Gawinowicz MA Zhao Y Colman DR (2001) The presynaptic particle web: ultrastructure, composition, dissolution, and reconstitution. Neuron 32:63–77. 11604139 10.1016/s0896-6273(01)00450-0

[CIT0082] Pierce A Unwin RD Evans CA Griffiths S Carney L Zhang L Jaworska E Lee CF Blinco D Okoniewski MJ Miller CJ Bitton DA Spooncer E Whetton AD (2008) Eight-channel iTRAQ enables comparison of the activity of six leukemogenic tyrosine kinases. Mol Cell Proteomics 7:853–863. 17951628 10.1074/mcp.M700251-MCP200

[CIT0083] Pirooznia M Wang T Avramopoulos D Valle D Thomas G Huganir RL Goes FS Potash JB Zandi PP (2012) SynaptomeDB: an ontology-based knowledgebase for synaptic genes. Bioinformatics 28:897–899. 22285564 10.1093/bioinformatics/bts040 PMC3307115

[CIT0084] Pittenger C Duman RS (2008) Stress, depression, and neuroplasticity: a convergence of mechanisms. Neuropsychopharmacology 33:88–109. 17851537 10.1038/sj.npp.1301574

[CIT0085] Porsolt RD Le Pichon M Jalfre M (1977) Depression: a new animal model sensitive to antidepressant treatments. Nature 266:730–732. 559941 10.1038/266730a0

[CIT0086] Ramakrishnan NA Drescher MJ Drescher DG (2012) The SNARE complex in neuronal and sensory cells. Mol Cell Neurosci 50:58–69. 22498053 10.1016/j.mcn.2012.03.009 PMC3570063

[CIT0087] Rathore SS Bend EG Yu H Hammarlund M Jorgensen EM Shen J (2010) Syntaxin N-terminal peptide motif is an initiation factor for the assembly of the SNARE-Sec1/Munc18 membrane fusion complex. Proc Natl Acad Sci U S A 107:22399–22406. 21139055 10.1073/pnas.1012997108 PMC3012463

[CIT0088] Ribrault C Reingruber J Petkovic M Galli T Ziv NE Holcman D Triller A (2011) Syntaxin1A lateral diffusion reveals transient and local SNARE interactions. J Neurosci 31:17590–17602. 22131420 10.1523/JNEUROSCI.4065-11.2011 PMC6623804

[CIT0089] Richmond JE Broadie KS (2002) The synaptic vesicle cycle: exocytosis and endocytosis in Drosophila and C. elegans. Curr Opin Neurobiol 12:499–507. 12367628 10.1016/s0959-4388(02)00360-4

[CIT0090] Rothman JE (1994) Mechanisms of intracellular protein transport. Nature 372:55–63. 7969419 10.1038/372055a0

[CIT0091] Rowland LM (2011) Who is resilient to depression? Multimodal imaging of the hippocampus in preclinical chronic mild stress model may provide clues. Biol Psychiatry 70:406–407. 21820531 10.1016/j.biopsych.2011.07.002

[CIT0092] Russo SJ Murrough JW Han MH Charney DS Nestler EJ (2012) Neurobiology of resilience. Nat Neurosci 15:1475–1484. 23064380 10.1038/nn.3234 PMC3580862

[CIT0093] Sakane A Manabe S Ishizaki H Tanaka-Okamoto M Kiyokage E Toida K Yoshida T Miyoshi J Kamiya H Takai Y Sasaki T (2006) Rab3 GTPase-activating protein regulates synaptic transmission and plasticity through the inactivation of Rab3. Proc Natl Acad Sci U S A 103:10029–10034. 16782817 10.1073/pnas.0600304103 PMC1502500

[CIT0094] Sanacora G Treccani G Popoli M (2012) Towards a glutamate hypothesis of depression: an emerging frontier of neuropsychopharmacology for mood disorders. Neuropharmacology 62:63–77. 21827775 10.1016/j.neuropharm.2011.07.036 PMC3205453

[CIT0095] Schimmoller F Simon I Pfeffer SR (1998) Rab GTPases, directors of vesicle docking. J Biol Chem 273:22161–22164. 9712825 10.1074/jbc.273.35.22161

[CIT0096] Schweizer FE Ryan TA (2006) The synaptic vesicle: cycle of exocytosis and endocytosis. Curr Opin Neurobiol 16:298–304. 16707259 10.1016/j.conb.2006.05.006

[CIT0097] Silinsky EM (2008) Selective disruption of the mammalian secretory apparatus enhances or eliminates calcium current modulation in nerve endings. Proc Natl Acad Sci U S A 105:6427–6432. 18420824 10.1073/pnas.0708814105 PMC2359788

[CIT0098] Snyder DA Kelly ML Woodbury DJ (2006) SNARE complex regulation by phosphorylation. Cell Biochem Biophys 45:111–123. 16679567 10.1385/CBB:45:1:111

[CIT0099] Sogaard M Tani K Ye RR Geromanos S Tempst P Kirchhausen T Rothman JE Sollner T (1994) A rab protein is required for the assembly of SNARE complexes in the docking of transport vesicles. Cell 78:937–948. 7923363 10.1016/0092-8674(94)90270-4

[CIT0100] Sousa N Lukoyanov NV Madeira MD Almeida OF Paula-Barbosa MM (2000) Reorganization of the morphology of hippocampal neurites and synapses after stress-induced damage correlates with behavioral improvement. Neuroscience 97:253–266. 10799757 10.1016/s0306-4522(00)00050-6

[CIT0101] Southwick SM Vythilingam M Charney DS (2005) The psychobiology of depression and resilience to stress: implications for prevention and treatment. Annu Rev Clin Psychol 1:255–291. 17716089 10.1146/annurev.clinpsy.1.102803.143948

[CIT0102] Sudhof TC (2004) The synaptic vesicle cycle. Annu Rev Neurosci 27:509–547. 15217342 10.1146/annurev.neuro.26.041002.131412

[CIT0103] Sudhof TC (2012) The presynaptic active zone. Neuron 75:11–25. 22794257 10.1016/j.neuron.2012.06.012 PMC3743085

[CIT0104] Sudhof TC Rothman JE (2009) Membrane fusion: grappling with SNARE and SM proteins. Science 323:474–477. 19164740 10.1126/science.1161748 PMC3736821

[CIT0105] Takai Y Sasaki T Shirataki H Nakanishi H (1996) Rab3A small GTP-binding protein in Ca(2+)-dependent exocytosis. Genes Cells 1:615–632. 9078389 10.1046/j.1365-2443.1996.00257.x

[CIT0106] Taliaz D Loya A Gersner R Haramati S Chen A Zangen A (2011) Resilience to chronic stress is mediated by hippocampal brain-derived neurotrophic factor. J Neurosci 31:4475–4483. 21430148 10.1523/JNEUROSCI.5725-10.2011 PMC6622886

[CIT0107] Togneri J Cheng YS Munson M Hughson FM Carr CM (2006) Specific SNARE complex binding mode of the Sec1/Munc-18 protein, Sec1p. Proc Natl Acad Sci U S A 103:17730–17735. 17090679 10.1073/pnas.0605448103 PMC1693815

[CIT0108] Toonen RF (2003) Role of Munc18-1 in synaptic vesicle and large dense-core vesicle secretion. Biochem Soc Trans 31: 848–850. 12887319 10.1042/bst0310848

[CIT0109] Van den Oever MC Goriounova NA Li KW Van der Schors RC Binnekade R Schoffelmeer AN Mansvelder HD Smit AB Spijker S De Vries TJ (2008) Prefrontal cortex AMPA receptor plasticity is crucial for cue-induced relapse to heroin-seeking. Nat Neurosci 11:1053–1058. 19160503 10.1038/nn.2165

[CIT0110] Vegh MJ de Waard MC van der Pluijm I Ridwan Y Sassen MJ van Nierop P van der Schors RC Li KW Hoeijmakers JH Smit AB van Kesteren RE (2012) Synaptic proteome changes in a DNA repair deficient ercc1 mouse model of accelerated aging. J Proteome Res 11:1855–1867. 22289077 10.1021/pr201203m

[CIT0111] Voets T Toonen RF Brian EC de Wit H Moser T Rettig J Sudhof TC Neher E Verhage M (2001) Munc18-1 promotes large dense-core vesicle docking. Neuron 31:581–591. 11545717 10.1016/s0896-6273(01)00391-9

[CIT0112] von Kriegstein K Schmitz F (2003) The expression pattern and assembly profile of synaptic membrane proteins in ribbon synapses of the developing mouse retina. Cell Tissue Res 311:159–173. 12596036 10.1007/s00441-002-0674-0

[CIT0113] Wang M Perova Z Arenkiel BR Li B (2014) Synaptic modifications in the medial prefrontal cortex in susceptibility and resilience to stress. J Neurosci 34:7485–7492. 24872553 10.1523/JNEUROSCI.5294-13.2014 PMC4035514

[CIT0114] Wang XC Li Q Jin X Xiao GH Liu GJ Liu NJ Qin YM (2015) Quantitative proteomics and transcriptomics reveal key metabolic processes associated with cotton fiber initiation. J Proteomics 114:16–27. 25449837 10.1016/j.jprot.2014.10.022

[CIT0115] Willner P Towell A Sampson D Sophokleous S Muscat R (1987) Reduction of sucrose preference by chronic unpredictable mild stress, and its restoration by a tricyclic antidepressant. Psychopharmacology (Berl) 93:358–364. 3124165 10.1007/BF00187257

[CIT0116] Wisniewski JR Zougman A Nagaraj N Mann M (2009) Universal sample preparation method for proteome analysis. Nat Methods 6:359–362. 19377485 10.1038/nmeth.1322

[CIT0117] Xie C Li J Guo T Yan Y Tang C Wang Y Chen P Wang X Liang S (2014) Rab3A is a new interacting partner of synaptotagmin I and may modulate synaptic membrane fusion through a competitive mechanism. Biochem Biophys Res Commun 444:491–495. 24472545 10.1016/j.bbrc.2014.01.090

[CIT0118] Xu HB Zhang RF Luo D Zhou Y Wang Y Fang L Li WJ Mu J Zhang L Zhang Y Xie P (2012) Comparative proteomic analysis of plasma from major depressive patients: identification of proteins associated with lipid metabolism and immunoregulation. Int J Neuropsychopharmacol 15:1413–1425. 22717272 10.1017/S1461145712000302

[CIT0119] Yang Y Yang D Tang G Zhou C Cheng K Zhou J Wu B Peng Y Liu C Zhan Y Chen J Chen G Xie P (2013) Proteomics reveals energy and glutathione metabolic dysregulation in the prefrontal cortex of a rat model of depression. Neuroscience 247: 191–200. 23727007 10.1016/j.neuroscience.2013.05.031

[CIT0120] Yu H Rathore SS Lopez JA Davis EM James DE Martin JL Shen J (2013) Comparative studies of Munc18c and Munc18-1 reveal conserved and divergent mechanisms of Sec1/Munc18 proteins. Proc Natl Acad Sci U S A 110:E3271–3280. 23918365 10.1073/pnas.1311232110 PMC3761595

[CIT0121] Zhan Y Yang YT You HM Cao D Liu CY Zhou CJ Wang ZY Bai SJ Mu J Wu B Zhan QL Xie P (2014) Plasma-based proteomics reveals lipid metabolic and immunoregulatory dysregulation in post-stroke depression. Eur Psychiatry 29:307–315. 24853294 10.1016/j.eurpsy.2014.03.004

[CIT0122] Zhang H Lu Y Luo B Yan S Guo X Dai J (2014) Proteomic analysis of mouse testis reveals perfluorooctanoic acid-induced reproductive dysfunction via direct disturbance of testicular steroidogenic machinery. J Proteome Res 13:3370–3385. 24940614 10.1021/pr500228d

[CIT0123] Zhou J Li J Li J Chen P Wang X Liang S (2010) Dried polyacrylamide gel absorption: a method for efficient elimination of the interferences from SDS-solubilized protein samples in mass spectrometry-based proteome analysis. Electrophoresis 31:3816–3822. 21064138 10.1002/elps.201000255

[CIT0124] Zhou J Bi D Lin Y Chen P Wang X Liang S (2012a) Shotgun proteomics and network analysis of ubiquitin-related proteins from human breast carcinoma epithelial cells. Mol Cell Biochem 359:375–384. 21853274 10.1007/s11010-011-1031-y

[CIT0125] Zhou K Yang Y Gao L He G Li W Tang K Ji B Zhang M Li Y Yang J Sun L Zhang Z Zhu H He L Wan C (2012b) NMDA receptor hypofunction induces dysfunctions of energy metabolism and semaphorin signaling in rats: a synaptic proteome study. Schizophr Bull 38:579–591. 21084551 10.1093/schbul/sbq132 PMC3329985

[CIT0126] Zilly FE Sorensen JB Jahn R Lang T (2006) Munc18-bound syntaxin readily forms SNARE complexes with synaptobrevin in native plasma membranes. PLoS Biol 4:e330. 17002520 10.1371/journal.pbio.0040330 PMC1570500

