# Supplementary material for: Quantitative Proteomic Analysis Reveals Molecular Adaptations in the Hippocampal Synaptic Active Zone of Chronic Mild Stress-Unsusceptible Rats
Source: Int J Neuropsychopharmacol. 2015 Sep 12;19(1):pyv100. doi: 10.1093/ijnp/pyv100 (PMC4772275; doi:10.1093/ijnp/pyv100)
Supplement: supplementary Methods [file ijnp_pyv100_index.html]

Supplementary Data | International Journal of Neuropsychopharmacology

## Supplementary Data

Data files

- Supplementary Data - Supplementary Data
- Supplementary Data - Supplementary Data
- Supplementary Data - Supplementary Data
- Supplementary Data - Supplementary Data
