# Supplementary material for: Quantitative Proteomic Analysis Reveals Molecular Adaptations in the Hippocampal Synaptic Active Zone of Chronic Mild Stress-Unsusceptible Rats
Source: Int J Neuropsychopharmacol. 2015 Sep 12;19(1):pyv100. doi: 10.1093/ijnp/pyv100 (PMC4772275; doi:10.1093/ijnp/pyv100)
Supplement: supplementary Methods [file pyv100Supplementary_Methods.docx]

**SUPPLEMENTARY METHODS**

**Animal Subjects and CMS Protocol**

Eighty-one healthy male Sprague-Dawley rats were purchased from the Animal Facility of Chongqing Medical University (Chongqing, China). Animal weight was approximately 165 g when adaptation for sucrose consumption was initiated and approximately 250 g at the start of the CMS protocol. All rats were singly housed and received a standard rodent diet and tap water ad libitum under a 12-hour–light–dark cycle (lights from 7:30 am to 7:30 pm) at a constant temperature of 21 to 22°C and humidity of 55 ± 5% except when one of these parameters was changed due to the stress regime. All animal care and treatment procedures were in accordance with the National Institutes of Health Guidelines for Animal Research (Guide for the Care and Use of Laboratory Animals). Moreover, all rats were monitored on daily basis.

The rats were allowed 1 week to acclimate to the environment and then given both 1% sucrose solution and water for 5 weeks for sucrose habituation. During this period, sucrose preference of all rats was tested twice weekly during the first 3 weeks and once weekly during the last 2 weeks. According to the sucrose intakes of the 3 final baseline tests, the rats were divided randomly into 2 groups and placed in separate rooms. Stress groups were exposed to a 4-week CMS procedure, whereas controls were left undisturbed.

The CMS protocol was performed according to the procedure described in our previous studies ([Willner et al., 1987](#_ENREF_13); [Grippo et al., 2006](#_ENREF_3); [Hu et al., 2013](#_ENREF_4); [Yang et al., 2013](#_ENREF_16)). In brief, rats were subjected to a variety of mild stressors: paired housing, 45^o^ cage tilt along the vertical axis, soiled cage (300 mL water spilled into bedding), exposure to an empty water bottle immediately following a period of acute water deprivation, stroboscopic illumination (300 flashes/min), continuous overnight illumination, and white noise. Details of the procedure including time and length of stressors are described in Fig. 1*a*. At the end of every week, the sucrose preference of all rats was assessed, and the OFT was performed in the last week.

**SPT**

The SPT was conducted to measure the anhedonic effect of CMS. A 2-bottle SPT was used, where all rats got access to both water and a 1 % sucrose solution for 24 hours during a no-stress period. The position of the 2 bottles (left/right sides of the cages) was randomly varied. All fluid consumption was recorded by weighing the 2 bottles before testing and after 24 hours. The sucrose preference was calculated according to the following formula: sucrose preference = sucrose intake (g)/(sucrose intake (g) + water intake (g)).

**OFT and FST**

Rats were placed in the testing room 1 hour before testing. The tests took place in a soundproof room between 8:00 and 12:00 am, unless otherwise noted. After each test, rats were returned to their home cages and then to the holding room once all animals were tested.

The OFT was carried out as previously described ([Yang et al., 2013](#_ENREF_16)). The open field apparatus, consisting of a black square cage measuring 100 × 100 × 40 cm^3^, was divided into sixteen 25 × 25 cm^2^ squares on the floor of the arena by a video-computerized tracking system (SMART, Panlab SL, Barcelona, Spain). The peripheral sector consisted of the squares close to the wall, and the central sector consisted of the 4 central squares. A single rat was gently placed in the center of the chamber, and after 30 seconds of adaptation, the locomotor activity (total distance in squares traversed), rearing number (posture sustained with hindpaws on the floor), and central activity (the time spent in the central sector) were recorded for 5 minutes using the SMART system. After each trial, the apparatus was thoroughly cleaned.

The FST was performed as described previously with slight modifications ([Porsolt et al., 1977](#_ENREF_9)). The rats were placed individually in Plexiglas cylinders (40 cm in height, 20 cm in diameter) filled with water (24 ± 1°C) up to a height of 30 cm. A 15-minute pretest period was followed 24 hours later by a 5-minute test period during which the total immobility time was recorded. The test was monitored by a video surveillance system. Water in the cylinders was changed before each trial.

**Preparation of Hippocampal Synaptic Junctions**

Synaptic junction-enriched fractions were obtained as previously reported ([Phillips et al., 2001](#_ENREF_7); [Abul-Husn et al., 2009](#_ENREF_1); [Hu et al., 2013](#_ENREF_4)). Briefly, rats were sacrificed by decapitation, and their brains were rapidly removed. The fresh hippocampal tissue samples were collected and pooled. Using a motor-operated Teflon-glass grinder, the pooled tissues were homogenized on ice in solution A (0.32 M sucrose, 1 mM MgCl_2_, and 0.1 mM CaCl_2_) containing a protease inhibitor cocktail (Sigma-Aldrich, St. Louis, MO). All of the following fractionation steps were carried out at 4°C unless otherwise specified. The Hom were centrifuged at 1400 *g* for 10 minutes, and the resultant pellet was then homogenized again. The second centrifugation was performed at 710 *g* for 10 minutes. The supernatants were pooled and centrifuged at 12 000 *g* for 30 minutes. The resulting pellet was resuspended in solution B (0.32 M sucrose and 0.1 mM CaCl_2_) then layered over a 1.2 M/1.0 M/0.85 M sucrose gradient (10 mL each). After ultracentrifugation (himac cp 80 wx, Hitachi Koki, Japan) at 82 × 500 *g* for 2 hours, the Syn from the 1.2 M/1.0 M interface was collected. To obtain synaptic junctions, an aliquot solution (synaptosomal fraction) was diluted with ice-cold 0.1 mM CaCl_2_ and brought to an equal volume of 2×hypotonic buffer (40 mM Tris-HCl pH6, 2% Triton X-100 [TX-100], 0.2 mM CaCl_2_) containing protease and phosphatase inhibitor cocktails. This solution was mixed by inversion and incubated on a shaker for 20 minutes at 4°C. The synaptic junction pellet were collected by centrifugation at 40 000 *g* for 20 minutes at 4°C. The supernatant, the Ves, was precipitated in acetone at -20°C overnight, and the resulting pellet was then air-dried. The resulting 4 fractions were analyzed by Western blot with antibodies to synaptophysin (ab52636, Abcam), syntaxin-1A (Stx1a, ab41453, Abcam), and PSD95 (#3450, CST). The following iTRAQ-labeling proteomic experiment was conducted on 6 sample pools corresponding to the 3 groups for biological replicates with each pool originated from 6 animals. The pools from the remaining 7 rats were used for subsequent Western-blot analysis.

**Extraction and Digestion of Synaptic Junctional Proteins**

The synaptic junction pellet was dissolved using a sample buffer (4% SDS, 10 mM DTT, 150 mM Tris-HCl, pH 8.0). The lysate was boiled in water for 5 minutes and then centrifuged at 40 000 *g* for 15 minutes. Protein concentrations were determined using a bicinchoninic acid protein assay kit according to the manufacturer’s instructions (Pierce). Bovine serum albumin was used as the standard. Subsequent protein digestion was performed in 10-kDa molecular weight cut-off centrifugation ﬁlters (Sartorius) ([Wisniewski et al., 2009](#_ENREF_14)). The sample was diluted with 200 μL of UA buffer (8 M urea, 150 mM Tris-HCl, pH 8.0) and then centrifuged at 14 000 *g* for 30 minutes. This step was repeated once. Then, 100 μL of 50 mM iodoacetamide in UA buffer was added to the filters, and the samples were incubated in darkness for 30 minutes. Filters were washed twice with 100 μL of UA buffer followed by 2 washes with 100 μL of dissolution buffer (50 mM triethylammoniumbicarbonate, pH 8.5). Proteins were digested overnight in 40 μL of dissolution buffer using trypsin (Promega) at an enzyme-to-protein ratio of 1:50 at 37°C. The released peptides were collected by centrifugation at 14 000 *g* for 10 minutes followed by 2 washes with dissolution buffer.

**iTRAQ Labeling and SCX Fractionation**

The tryptic peptides were labeled with an iTRAQ-4plex kit (Applied Biosystems) according to the manufacturer’s protocol. Samples from the control group were labeled with reagents 115 and 116, samples from the susceptible group were labeled with reagents 114 and 116, and samples from the unsusceptible group were labeled with reagents 114 and 115. Two independent experiments were performed for biological replicates. Incubation was allowed to proceed at room temperature for 2 hours and then stopped by addition of 10 mM KH_2_PO_4_ in 25% acetonitrile (pH 3.0). Subsequently, all 3 labeled-samples were pooled, vacuum-dried, and further fractionated offline using SCX chromatography. Briefly, the peptides were dissolved and loaded onto a polysulfoethyl 4.6 × 100 mm column (5 µm, 200 Å, PolyLC Inc.) at a flow rate of 1 mL/min. A suitable gradient elution was applied to separate peptides at a flow rate of 1 mL/min with elution buffer (10 mM KH_2_PO_4_, 500 mM KCl in 25% acetonitrile, pH 3.0). The resulting 30 fractions were combined to 10 pools and desalted on C18 Cartridges (Empore SPE Cartridges C18 [standard density], bed I.D. 7 mm, volume 3 mL, Sigma, St. Louis, MO). Each final fraction was concentrated by a vacuum concentrator and reconstituted with 40 µL of 0.1% formic acid for LC-MS/MS analysis.

**Light Chromatography Tandem Mass Spectrometry Analysis**

The SCX fractions were analyzed using a TripleTOF 5600 mass spectrometer (MS; AB SCIEX) equipped with a splitless nanoLC-Ultra 2D plus system and a cHiPLC Nanoflex microchip system (Eksigent, Dublin, CA). The Nanoflex system uses replaceable microfluidic traps and columns packed with ChromXP C18 (3 μm, 120 Å) for online trapping, desalting, and analytical separations. The sample was loaded, and trapping and desalting were performed at 2 μL/min for 10 minutes with a 100% mobile phase A (2% acetonitrile/0.2% formic acid/98% water). For peptide elution, the gradient started with a 5% mobile phase B (98% acetonitrile/0.2% formic acid/2% water) and linearly progressed to 24% over 70 minutes at a flow rate of 300 nL/min. The total gradient length was 120 minutes. MS data acquisition was performed in the information dependent acquisition mode. For MS scans, the TripleTOF 5600 MS was operated with a resolving power of 30000 FWHM. Information dependent acquisition survey scans were acquired in 250 milliseconds with mass range of m/z 350 to 1250. Specifically, 30 × 100 ms MS/MS were followed at a 120-cps (counts/s) trigger with a precursor charge state of +2 to +5. Dynamic exclusion was set for 18 seconds. Collision energies were calculated on-the-fly for all precursor ions using empirical equations based on mass and charge, and the Enhance iTRAQ function was turned on to improve the efficiency of the collision-induced dissociation.

**Protein Identification and Quantitation**

Protein identification and iTRAQ quantitation were performed with ProteinPilot 4.5 software (AB SCIEX) using the Paragon algorithm (4.5.0.0.1654) as the search engine. All data were searched using a publicly available rat UniProt release 2013_03 database of 41 766 protein sequences. The user-defined search parameters included iTRAQ 4-plex quantification, trypsin digestion, selection only of tryptic peptides with 2 missed cleavages, variable modifications of methionine oxidation, fixed modification of carbamidomethyl cysteine, Triple TOF 5600, thorough searching mode, peptide mass tolerance of ±20 ppm, and fragment mass tolerance of 0.1 Da, and a minimum protein threshold of 95% confidence. Identified proteins were grouped by the ProGroup algorithm (AB SCIEX) to minimize redundancy. Peptides that matched multiple proteins were not included in the software quantification. To minimize false positive results, a strict cutoff for protein identification was applied with the unused protein score ≥1.3, which corresponds to a confidence limit of 95%, and at least one unique peptide with 95% confidence were considered for protein identification as listed in supplementary Table 1. For the false discovery rate (FDR) calculation, an automatic decoy database search strategy was employed to estimate FDR using the PSPEP (Proteomics System Performance Evaluation Pipeline Software, integrated in the ProteinPilot Software). FDR was defined as the percentage of decoy proteins identified against the total protein identification. All reported data were based on FDR <1% confidence for protein identification as determined by ProteinPilot. A significance threshold of 95% was chosen as a criterion for each individual experiment. Redundant proteins and peptides as well as proteins identified by reverse sequence were removed from the list. For this study, proteins with <3 unique peptides (95% confidence) or having an error factor >2.0 were eliminated to improve the confidence of protein quantitation ([Vegh et al., 2012](#_ENREF_11)). Peptide summaries were exported from ProteinPilot and isotope correction and relative quantification was calculated. The final ratios were calculated from the average of the ratios obtained from the 2 independent experiments. In each experiment, bias correction for unequal mixing in the differently-labeled samples was performed based on the assumption that most proteins do not change in expression. Thus, if the sample from each experimental condition was not combined in exactly equal amounts, this bias correction would fix the systematic error. The software identifies the median average protein ratio and corrects it to unity and then applies this factor to all quantification results. The results were then exported into Microsoft Excel for manual data interpretation. For quantitative analysis, observed proteins with iTRAQ ratios of >1.2 and <0.83 were considered to be differentially expressed, as used in previous studies ([Lin et al., 2013](#_ENREF_6); [Zhang et al., 2014](#_ENREF_17); [Wang et al., 2015](#_ENREF_12)). All raw and metadata of the proteome have been deposited to the ProteomeXchange Consortium via the PRIDE partner repository with the dataset identifier PXD002540.

**Bioinformatics Analysis**

The subcellular location and function of the identified differential proteins were elucidated by the gene ontology component and functional terms, respectively. The gene ontology annotation associated with a given gene list was obtained using the Universal Protein Resource (UniProt) (http://www. uniprot. org) and the Database for Annotation, Visualization, and Integrated Discovery (DAVID) 6.7 Bioinformatics tool (http://david.abcc.ncifcrf.gov/) ([Huang da et al., 2009](#_ENREF_5)). More detailed descriptions of these differential membrane proteins was derived from SynaptomeDB (http://psychiatry.igm.jhmi.edu/SynaptomeDB/) ([Pirooznia et al., 2012](#_ENREF_8)). Furthermore, the identified proteins involved in membrane trafficking were mapped to the protein interaction network, and the Search Tool for the Retrieval of Interacting Gene/Proteins (STRING) (http://string.embl.de/) was used to qualify the physical and functional interactions of these proteins. The confidence score that defined interaction confidence was set at ≥0.7 (high confidence) ([Zhou et al., 2012](#_ENREF_18)).

**Antibodies and Western-Blot Analyses**

Western blots were first performed on the 3 synaptic junction protein extracts that were also used for the iTRAQ experiments. In addition, by immunoblotting, we analyzed 3 independent samples from pools of 7 animals kept under the identical experimental conditions as the animals for iTRAQ experiments. At the same time, the additional protein extracts from the aforementioned Hom, Syn, or Ves fractions were also used for Western-blot detection. The procedures of electrophoresis, transfer and immunodetection were performed according to our previous study ([Xu et al., 2012](#_ENREF_15); [Hu et al., 2013](#_ENREF_4)). The primary antibodies used were as follows: Rab3a (ab3335, 1:2000); Stxbp1 (Munc18-1, ab124920, 1:4000); Syn1 (ab18814, 1:1000); Stx1a (ab41453, 1:3000); SNAP25 (ab5666, 1:4000); VAMP2 (ab3347, 1:2000) (all purchased from Abcam); Syt1 (Millipore MAB5200, 1:1000); and Stx1b (Synaptic Systems 110402, 1:1000). Horseradish peroxidase-conjugated anti-mouse and anti-rabbit IgG (purchased from Bio-Rad, dilution 1:15 000) were used as secondary antibodies. After immunodetection, the intensity of the immunostained bands were normalized for the total protein intensities measured by Coomassie blue from the same blot ([Van den Oever et al., 2008](#_ENREF_10); [Counotte et al., 2010](#_ENREF_2)). The images were subjected to densitometric analysis performed using Quantity One Software (Bio-Rad).

**Statistical Analysis**

The statistical analyses were carried out using SPSS 16.0 ([Hu et al., 2013](#_ENREF_4); [Yang et al., 2013](#_ENREF_16)). The data from the SPT were analyzed by repeated measurement ANOVA factoring treatment (control, susceptible, and unsusceptible) and time point (baseline and weeks 1, 2, 3, and 4). To detect significant differences between the experimental groups and time points, MANOVAs and Bonferroni post-tests were used. The OFT and FST data were analyzed by 1-way ANOVA followed by posthoc LSD test. Exceptions were the central activity parameters of OFT, which displayed a nonnormal distribution that was analyzed by nonparametric tests (the Kruskal-Wallis test followed by the Mann–Whitney test). In addition, the data from Western blots of protein expression were compared using Student’s *t* tests. A *P* value of <.05 was considered to be statistically significant. Statistics were presented as means ± SE.

**REFERENCES**

Abul-Husn NS, Bushlin I, Moron JA, Jenkins SL, Dolios G, Wang R, Iyengar R, Ma'ayan A, Devi LA (2009) Systems approach to explore components and interactions in the presynapse. Proteomics 9:3303–3315.

Counotte DS, Li KW, Wortel J, Gouwenberg Y, Van Der Schors RC, Smit AB, Spijker S (2010) Changes in molecular composition of rat medial prefrontal cortex synapses during adolescent development. Eur J Neurosci 32:1452–1460.

Grippo AJ, Beltz TG, Weiss RM, Johnson AK (2006) The effects of chronic fluoxetine treatment on chronic mild stress-induced cardiovascular changes and anhedonia. Biol Psychiatry 59:309–316.

Hu Y, Zhou J, Fang L, Liu H, Zhan Q, Luo D, Zhou C, Chen J, Li Q, Xie P (2013) Hippocampal synaptic dysregulation of exo/endocytosis-associated proteins induced in a chronic mild-stressed rat model. Neuroscience 230:1–12.

Huang da W, Sherman BT, Lempicki RA (2009) Systematic and integrative analysis of large gene lists using DAVID bioinformatics resources. Nat Protoc 4:44–57.

Lin XQ, Liang SL, Han SY, Zheng SP, Ye YR, Lin Y (2013) Quantitative iTRAQ LC-MS/MS proteomics reveals the cellular response to heterologous protein overexpression and the regulation of HAC1 in Pichia pastoris. J Proteomics 91:58–72.

Phillips GR, Huang JK, Wang Y, Tanaka H, Shapiro L, Zhang W, Shan WS, Arndt K, Frank M, Gordon RE, Gawinowicz MA, Zhao Y, Colman DR (2001) The presynaptic particle web: ultrastructure, composition, dissolution, and reconstitution. Neuron 32:63–77.

Pirooznia M, Wang T, Avramopoulos D, Valle D, Thomas G, Huganir RL, Goes FS, Potash JB, Zandi PP (2012) SynaptomeDB: an ontology-based knowledgebase for synaptic genes. Bioinformatics 28:897–899.

Porsolt RD, Le Pichon M, Jalfre M (1977) Depression: a new animal model sensitive to antidepressant treatments. Nature 266:730–732.

Van den Oever MC, Goriounova NA, Li KW, Van der Schors RC, Binnekade R, Schoffelmeer AN, Mansvelder HD, Smit AB, Spijker S, De Vries TJ (2008) Prefrontal cortex AMPA receptor plasticity is crucial for cue-induced relapse to heroin-seeking. Nat Neurosci 11:1053–1058.

Vegh MJ, de Waard MC, van der Pluijm I, Ridwan Y, Sassen MJ, van Nierop P, van der Schors RC, Li KW, Hoeijmakers JH, Smit AB, van Kesteren RE (2012) Synaptic proteome changes in a DNA repair deficient ercc1 mouse model of accelerated aging. J Proteome Res 11:1855–1867.

Wang XC, Li Q, Jin X, Xiao GH, Liu GJ, Liu NJ, Qin YM (2015) Quantitative proteomics and transcriptomics reveal key metabolic processes associated with cotton fiber initiation. J Proteomics 114:16–27.

Willner P, Towell A, Sampson D, Sophokleous S, Muscat R (1987) Reduction of sucrose preference by chronic unpredictable mild stress, and its restoration by a tricyclic antidepressant. Psychopharmacology (Berl) 93:358–364.

Wisniewski JR, Zougman A, Nagaraj N, Mann M (2009) Universal sample preparation method for proteome analysis. Nat Methods 6:359–362.

Xu HB, Zhang RF, Luo D, Zhou Y, Wang Y, Fang L, Li WJ, Mu J, Zhang L, Zhang Y, Xie P (2012) Comparative proteomic analysis of plasma from major depressive patients: identification of proteins associated with lipid metabolism and immunoregulation. Int J Neuropsychopharmacol 15:1413–1425.

Yang Y, Yang D, Tang G, Zhou C, Cheng K, Zhou J, Wu B, Peng Y, Liu C, Zhan Y, Chen J, Chen G, Xie P (2013) Proteomics reveals energy and glutathione metabolic dysregulation in the prefrontal cortex of a rat model of depression. Neuroscience 247:191–200.

Zhang H, Lu Y, Luo B, Yan S, Guo X, Dai J (2014) Proteomic analysis of mouse testis reveals perfluorooctanoic acid-induced reproductive dysfunction via direct disturbance of testicular steroidogenic machinery. J Proteome Res 13:3370–3385.

Zhou J, Bi D, Lin Y, Chen P, Wang X, Liang S (2012) Shotgun proteomics and network analysis of ubiquitin-related proteins from human breast carcinoma epithelial cells. Mol Cell Biochem 359:375–384.
